# *Brucella* spp. of amphibians comprise genomically diverse motile strains competent for replication in macrophages and survival in mammalian hosts

**DOI:** 10.1038/srep44420

**Published:** 2017-03-16

**Authors:** Sascha Al Dahouk, Stephan Köhler, Alessandra Occhialini, María Pilar Jiménez de Bagüés, Jens Andre Hammerl, Tobias Eisenberg, Gilles Vergnaud, Axel Cloeckaert, Michel S. Zygmunt, Adrian M. Whatmore, Falk Melzer, Kevin P. Drees, Jeffrey T. Foster, Alice R. Wattam, Holger C. Scholz

**Affiliations:** 1 German Federal Institute for Risk Assessment (BfR), Department of Biological Safety, Berlin, Germany; 2 RWTH Aachen University, Department of Internal Medicine III, Aachen, Germany; 3 Université Montpellier, Centre d’études d’agents Pathogènes et Biotechnologies pour la Santé (CPBS), Montpellier, France; 4 CNRS, FRE3689, CPBS, Montpellier, France; 5 Unidad de Producción y Sanidad Animal, Centro de Investigación y Tecnología Agroalimentaria, Instituto Agroalimentario de Aragón – IA2 (CITA-Universidad de Zaragoza), Zaragoza, Spain; 6 Landesbetrieb Hessisches Landeslabor, Gießen, Germany; 7 I2BC, CNRS, CEA, Univ. Paris-Sud, Université Paris-Saclay, Gif-sur-Yvette, France; 8 ISP, INRA, Université François Rabelais de Tours, UMR1282, Nouzilly, France; 9 Animal & Plant Health Agency, Addlestone, United Kingdom; 10 Friedrich-Loeffler-Institut, German National Reference Laboratory for Animal Brucellosis, Jena, Germany; 11 University of New Hampshire, Department of Molecular, Cellular, and Biomedical Sciences, Durham, NH, USA; 12 Biocomplexity Institute, Virginia Tech, Blacksburg, VA, USA; 13 Bundeswehr Institute of Microbiology and German Center for Infection Research (DZIF), Munich, Germany

## Abstract

Twenty-one small Gram-negative motile coccobacilli were isolated from 15 systemically diseased African bullfrogs (*Pyxicephalus edulis*), and were initially identified as *Ochrobactrum anthropi* by standard microbiological identification systems. Phylogenetic reconstructions using combined molecular analyses and comparative whole genome analysis of the most diverse of the bullfrog strains verified affiliation with the genus *Brucella* and placed the isolates in a cluster containing *B. inopinata* and the other non-classical *Brucella* species but also revealed significant genetic differences within the group. Four representative but molecularly and phenotypically diverse strains were used for *in vitro* and *in vivo* infection experiments. All readily multiplied in macrophage-like murine J774-cells, and their overall intramacrophagic growth rate was comparable to that of *B. inopinata* BO1 and slightly higher than that of *B. microti* CCM 4915. In the BALB/c murine model of infection these strains replicated in both spleen and liver, but were less efficient than *B. suis* 1330. Some strains survived in the mammalian host for up to 12 weeks. The heterogeneity of these novel strains hampers a single species description but their phenotypic and genetic features suggest that they represent an evolutionary link between a soil-associated ancestor and the mammalian host-adapted pathogenic *Brucella* species.

The genus *Brucella*, established in 1920 by Meyer and Shaw, currently comprises twelve species^1^ (http://www.bacterio.cict.fr/b/brucella.html) that are historically differentiated by host tropism, pathogenicity and phenotypic traits. A decade ago the genus consisted of six “classical” *Brucella* species (*B. melitensis, B. abortus, B. suis, B. canis, B. ovis*, and *B. neotomae*), which are also referred to as the “core” *Brucella*. Three of these species (*B. melitensis, B. abortus* and *B. suis*) are important zoonotic pathogens that infect humans and a variety of other mammals, causing a disease called brucellosis. This is considered to be one of the most important zoonoses worldwide, with 500,000 human cases reported each year^2^. Due to its low infectious dose and ease of transmission as an aerosol, *Brucella* was one of the first microorganisms weaponized by the U.S. military in 1950s^3^ and is listed as a category B bioterrorism agent by the U.S. Centers for Disease Control and Prevention (CDC) and the National Institute of Allergy and Infectious Diseases (NIAID)^4^.

The group of classical *Brucella* species was extended in 2007 to include *B. ceti* and *B. pinnipedialis*, isolated from marine mammals^5^. The first phenotypically atypical species, *B. microti*, was initially isolated from common voles^6^,^7^ and then from soil^8^. *B. microti* is different from the classical species in that it is fast-growing and metabolically very active, resembling *Ochrobactrum*, the closest phylogenetic neighbour of *Brucella*^7^. Although metabolically different, the genome sequence of *B. microti* (strain CCM 4915^T^) is nearly identical to *B. suis* 1330^9^, making it appear that its phenotypic differences may reflect differential gene regulation^10^. Subsequent whole genome analyses, however, indicate that, while *B. microti* is closely related to the other core *Brucella*, it represents a distinct basal lineage^11^. Most recently, *B. papionis*, isolated from baboons, phylogenetically clustering with the core *Brucella* spp.^12^,^13^, and *B. vulpis* from red foxes, forming a long-branched sister clade to the classical species^1^,^14^, were described.

Additional atypical *Brucella* isolates have been described. The first, *B. inopinata* (strain BO1), was isolated from a breast implant wound infection^15^,^16^. This strain showed lower sequence similarities in most housekeeping genes^15^ and differed at 5 nucleotide positions in the 16S rRNA sequence from the one sequence shared by all core *Brucella*^16^. Metabolically, *B. inopinata* (BO1) is as active as *B. microti*^7^,^16^. This finding was followed by a second human isolate (BO2) from a patient with chronic destructive pneumonia that revealed an identical 16S rRNA gene sequence to *B. inopinata* (BO1)^17^. In addition, the phylogenetic comparison of novel atypical strains isolated from rodents in Australia showed that these, along with the BO2 strain and *B. inopinata* (BO1), are united in a clade that is separate from the core *Brucella* spp.^18^.

In the past few years, more and more *Brucella* isolates have been reported from cold-blooded hosts, i.e. African bullfrogs (*Pyxicephalus edulis*)^19^, big-eyed tree frogs (*Leptopelis vermiculatus*)^20^, White’s tree frog^21^, and Pacman frog (*Ceratophrys ornata*)^22^. At present, the pathogenicity of these newly described species and atypical strains for humans is unknown but warrants investigation. Human pathogenicity cannot be excluded because human infections with *B. inopinata* (BO1) and strain BO2 described as *B. inopinata*-like have been reported^15^,^17^.

The present work is a comprehensive molecular and phenotypic study of a large collection of isolates from African bullfrogs building on a previous preliminary description of two of these strains^19^. We conducted an in-depth genome analysis and also evaluated the virulence of the potentially new *Brucella* species for mammalian hosts both in *in vitro* and *in vivo* models of infection.

## Results

Historically, CO_2_ requirement, H_2_S production, urea hydrolysis, agglutination with monospecific sera, dye sensitivity, and phage lysis are determined for the identification and sub-differentiation of *Brucella* spp. To gain deeper insight into the composition of this novel group of amphibian *Brucella* sp., all currently available bullfrog isolates have been characterized using a comprehensive combination of molecular and classical microbiological approaches^10^.

### Analysis of phenotypic characteristics

Primary isolation from various tissues of dead or moribund African bullfrogs revealed Gram-negative coccoid rods that were identified as *O. anthropi* by API^®^-20NE and Vitek2^®^ Compact (bioMérieux, Marcy-l’Étoile, France)^19^. The bacterial isolates showed nonfastidious rapid growth on trypticase soy agar (TSA), sheep blood agar, and standard nutrient agar at 25–42 °C and also grew on MacConkey agar. On Columbia agar, colonies did not display haemolysis. All 21 strains could be easily cultured on *Brucella* agar at 37 °C without supplementary CO_2_. After 24 h, typical raised, convex and circular colonies, 1–2 mm in diameter, were visible. Seventeen of these strains produced translucent to whitish colonies, but four strains (09RB8913, 09RB8914, 09RB8915, and 09RB8918) developed colonies with a markedly brownish pigmentation.

All strains were both catalase- and oxidase-positive, raising suspicion of *Brucella*. They produced H_2_S, and urea was hydrolyzed within 10 to 45 min by all but two of the strains. Strains 10RB9215 and 10RB9213 had a prolonged hydrolysis time of 80 and 90 min, respectively, revealing reduced urease activity (Table 1). All isolates were able to grow in the presence of thionine at dilutions of 1/25,000, 1/50,000, and 1/100,000 and in the presence of basic fuchsin at dilutions of 1/50,000 and 1/100,000.

Spontaneous agglutination could not be induced by trypaflavine, and ruby coloured colonies were not visible after staining with crystal violet. In addition, none of the strains agglutinated with monospecific anti-R (rough) serum. All the bullfrog isolates presented a smooth lipopolysaccharide phenotype, but none agglutinated with either anti-M or anti-A monospecific sera. In three of the strains (10RB9215, 10RB9211 and 10RB9212) a slight agglutination was seen using high concentrations (1:10 or 1:20) of anti-A sera.

The bullfrog isolates were not lysed by the bacteriophages F1, F25, Tb, BK2, Iz, Wb, Fi, and R/C, neither at the routine test dilution (RTD) nor at 10^4^x RTD (Table 1).

The Micronaut^®^ BfR *Brucella* assay (Merlin Diagnostika) was used for biochemical profiling^23^ and the bullfrog strains generally displayed high metabolic activity, comparable to *B. inopinata* (BO1) and *B. microti*, but in contrast to the relatively low activity of classical *Brucella* species (Supplementary Table S1). A number of reactions considered to be typical for brucellae (except for *B. papionis*), i.e. H-hydroxyproline-βNA (HP: +), Glu(pNA)-OH (ENAOH: −), Pyr-pNA (PYRNA: −), suggest an allocation of the bullfrog strains to the genus *Brucella*. Hierarchical cluster analysis performed by the Ward’s linkage algorithm positioned the amphibian *Brucella* sp. strains between *B. inopinata* (BO1) and *B. microti*, close to *B. suis* biovars 1–4 and *B. canis* (Supplementary Fig. S1). The metabolic activity varied within the bullfrog isolates group (Supplementary Table S1).

**Figure 1:**
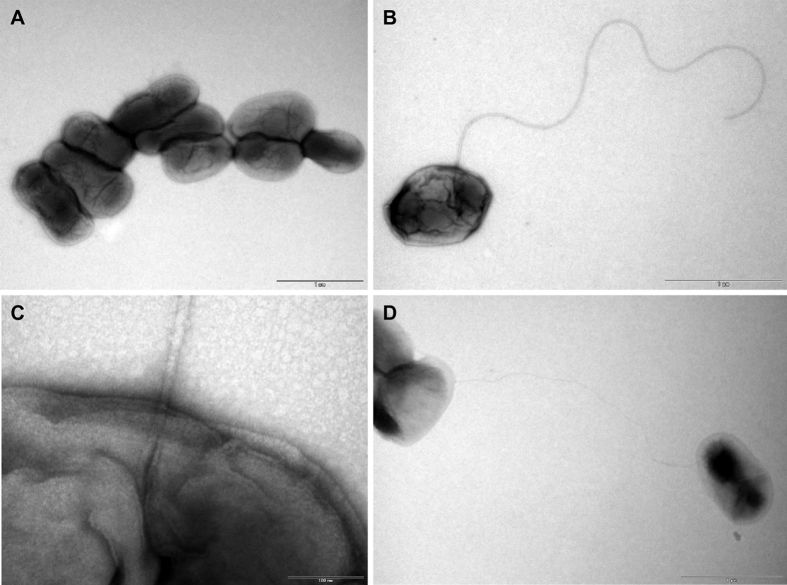
Electron micrographs of flagellated *Brucella* sp. isolated from African bullfrogs. Electron micrographs showing individual cells or clusters of strain no. 10RB9206 (**A**). Some bacteria were flagellated with a polar flagellum (**B**,**C**) and presented pili-like structures (**D**).

Semi-solid agar testing for bacterial swarming showed that the African bullfrog strains are motile (Supplementary Fig. S2A–D), in strong contrast to the classical brucellae like *B. melitensis* 16 M (Supplementary Fig. S2E). Some of the bullfrog isolates migrated farther through the agar than *O. anthropi* LMG 3331 (Supplementary Fig. S2F), others showed a similar migration pattern, e.g. 09RB8471. Transmission electron micrographs (40,000x) showed bacterial cells (strain 10RB9206) individually arranged or in irregular clusters, exhibiting a mean average diameter of 0.5 μm and a length of 1 μm (Fig. 1A). Some produced a polar sheathed flagellum (Fig. 1B,C). The expression of the flagellum *in vitro* increased from 12 to 84 h of culture. In addition, individual bacteria presented pili-like structures (Fig. 1D).

### Molecular analyses

In order to confirm the assignment of the African bullfrog strains to the genus and to refine their position within the known *Brucella* species a series of molecular typing methods with increasing resolution were applied.

#### *Bcsp31*, IS*711*, and Bruce-ladder multiplex PCR

The presence of the *Brucella*-specific IS*711* element and amplification of *bcsp31* using *Brucella* specific primers in all strains demonstrated their affiliation with the genus *Brucella*. Strains were identical in the Bruce-ladder PCR, a multiplex assay for the differentiation of *Brucella* species, displaying a previously known banding pattern with amplicon sizes of 152, 272, 450, 587, and 794 bp. IS*711* was also detected by Southern blot analysis. The IS*711* copy number varied from one copy to more than 14 copies depending on the genotype determined with other molecular analyses (*data not shown*).

#### 16S rRNA (*rrs*) and *recA* genes

Comparative sequence analysis of all 21 bullfrog isolates confirmed the two types of 16S rRNA gene sequences previously described^19^ (Table 2). Type A was identical with the sequence of *B. inopinata* whereas type B contained a 44 nt insertion apparently derived from *Ochrobactrum* species. A *recA*-gene based phylogenetic reconstruction revealed three main clusters and two strains with a unique sequence type each (Table 2).

#### MLSA and MLVA

**Figure 2:**
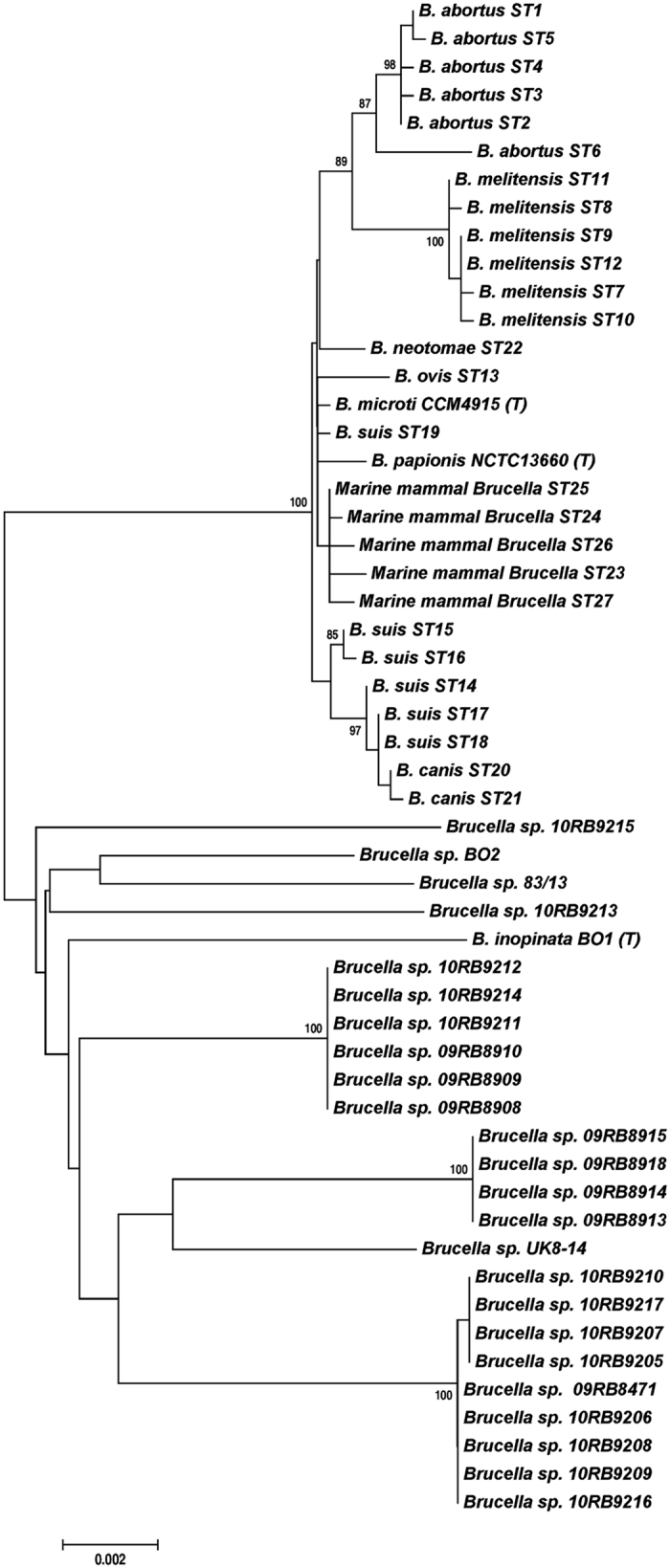
Phylogenetic analysis of the bullfrog isolates in comparison to *Brucella* spp. inferred by MLSA. Phylogenetic relationship of amphibian isolates with other *Brucella* species based on eight-locus MLSA. Numbers at nodes correspond to proportions of 500 resamplings that support the topology shown with only values >80% indicated. The bar indicates the number of substitutions per nucleotide position. UK8/14 represents a previously described amphibian isolate from the United Kingdom^21^.

Multilocus sequence analysis (MLSA) showed the amphibian isolates to be different from all previously described *Brucella* spp. and comprise five distinct lineages with two singleton isolates and three larger clusters labelled A through E (Table 2). Cluster E consists of two subtypes with a different sequence at only a single locus. With the exception of the *thyA* allele 1, seen in seven isolates representing genotypes B and C, all alleles were novel, having not been previously reported in any *Brucella* examined to date. Distinct novel alleles were also found in each of the five distinct lineages. The clustering of the African bullfrog strains is entirely consistent with other molecular typing approaches (Table 2). Phylogenetic placement relative to other isolates is shown in Fig. 2 and clearly illustrates the five distinct lineages comprising clusters of six identical isolates, four identical isolates and nine isolates varying only at a single locus and two singleton isolates (10RB9215 and 10RB9213). This analysis clearly shows that the isolates could all justifiably be characterized as members of the atypical *Brucella* sharing a similar relationship to “core *Brucella*” as do *B. inopinata* (BO1) and *B. inopinata*-like isolates (strain BO2, Australian rodent strains). A recently described amphibian isolate from the UK^21^ was included in the analysis and found to be related to, but distinct from, the isolates described here, being most closely related to genotype D isolates.

In the comparative multilocus variable-number tandem repeat (VNTR) analysis (MLVA), the African bullfrog strains grouped together with *B. inopinata* (BO1), forming a separate cluster within the genus *Brucella* (Supplementary Fig. S3). *Brucella neotomae, B. microti, B. suis* bv 5, and the marine mammal brucellae, *B. ceti* and *B. pinnipedialis*, were most closely related (Supplementary Fig. S4). By use of panel 1 VNTRs, the 21 bullfrog isolates clustered into five different genotypes (MLVA-8 genotypes 101–105 [ http://microbesgenotyping.i2bc.paris-saclay.fr/]) that have not yet been previously identified in more than 3,500 other *Brucella* strains. On the basis of the MLVA-11 data set, seven individual genotypes could be identified, numbered 196 to 202.

### Phylogenetic and comparative analyses

#### Phylogeny

**Figure 3:**
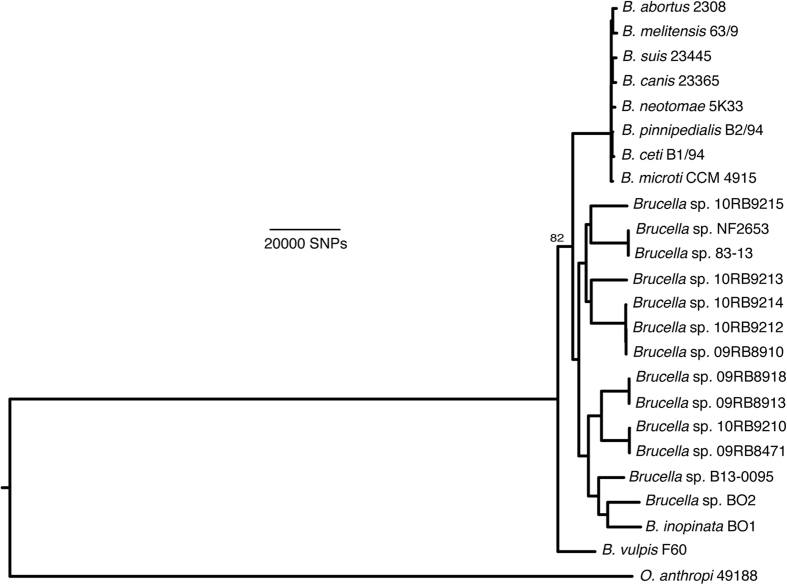
Phylogenetic placement of the African bullfrog strains based on whole genome sequencing data. Trees were constructed using maximum parsimony with nodal support for bootstrapping shown only for the branch that had less than 90% support within the basal clade containing the atypical *Brucella* strains. Strains NF2653 and 83-13 were isolated from Australian rodents.

Whole genome sequencing of nine isolates, at least two each from the three major clades and the two strains found in separate lineages, confirmed their affiliation with the genus *Brucella* (Fig. 3). Phylogenetically, the African bullfrog strains are basal to the classical *Brucella* species and are intermixed in the tree with isolates from Australian rodents and the unusual BO1 and BO2 isolates associated with human clinical disease but of unknown transmission route to humans. Four distinct clades of bullfrog isolates are apparent but limited bootstrap support for these basal groups and a high level of homoplasy (homoplasy index = 0.5091) that is substantially increased by inclusion of these lineages in phylogenies suggest substantial uncertainty for the topology of the amphibian and related *Brucella* strains.

The bullfrog isolates revealed a number of genomic regions that are not seen in any of the known *Brucella* genomes. These regions are either shared across all of the African bullfrog strains, shared across some of the strains, or found in a specific genome (Supplementary Table S2).

Since our phenotypic investigations revealed that none of the strains agglutinated with monospecific anti-*Brucella* sera and testing for bacterial swarming showed that they were motile, we focused on the *wbk* region involved in O-antigen synthesis and the flagellar genes.

#### LPS and O-antigen comparison

**Figure 4:**
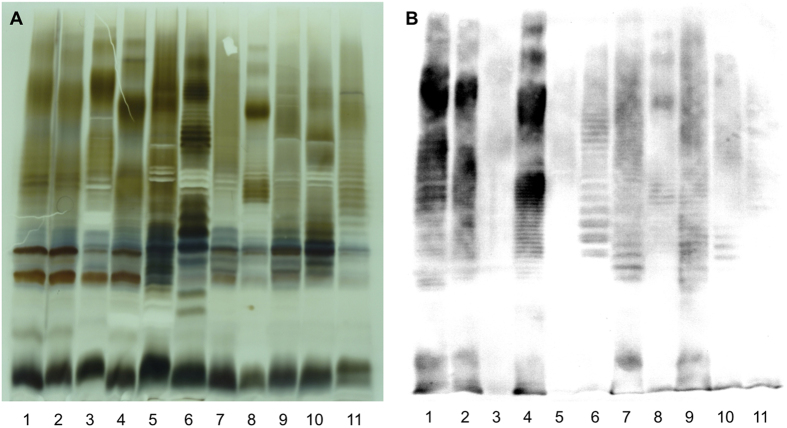
Electrophoretic lipopolysaccharide (LPS) profiles produced by the amphibian *Brucella* sp. strains. Silver staining (**A**) and Western blot (**B**) profiles with mAb A68/10A06/B11 (anti-R-LPS) after SDS-PAGE of proteinase K-digested S-LPS preparations of *B. melitensis* 16 M (M-dominant reference strain) (lanes 1), *B. suis* 1330 (A-dominant reference strain) (lanes 2), *B. microti* CCM 4915 (lanes 3), *Brucella* sp. strain 83/13 (wild rodent isolate from Australia) (lanes 4), *B. inopinata* BO1 (lanes 5), *Brucella* sp. strain BO2 (lanes 6), African bullfrog strains 09RB8910 (lanes 7), 10RB9207 (lanes 8), 10RB9215 (lanes 9), 10RB9213 (lanes 10), 09RB8915 (lanes 11).

SDS-PAGE and silver stain analyses revealed that all bullfrog isolates produce S-LPS, but with a profile clearly different from A- or M-dominant S-LPS of classical *Brucella* species (Fig. 4A). Five distinct S-LPS profiles were found correlating with the genotypes assessed by molecular analyses. None of these five unique profiles corresponded to the atypical S-LPS profile of BO2. Furthermore, none of the bullfrog isolates had a positive ELISA when mAbs against the different classical *Brucella* O-chain epitopes were used (Fig. 4B), which is similar to BO2. There was a weak agglutination with anti-A polyclonal serum, indicating that the binding may be nonspecific. Nevertheless, all isolates reacted with anti-R-LPS mAb A68/10A6/B11 in Western blot revealing both the R-LPS moiety and S-LPS part as in previous studies^24^,^25^ (Fig. 4B), suggesting that the core-lipid A of the amphibian *Brucella* sp. strains is structurally related to that of classical *Brucella* species.

**Figure 5:**
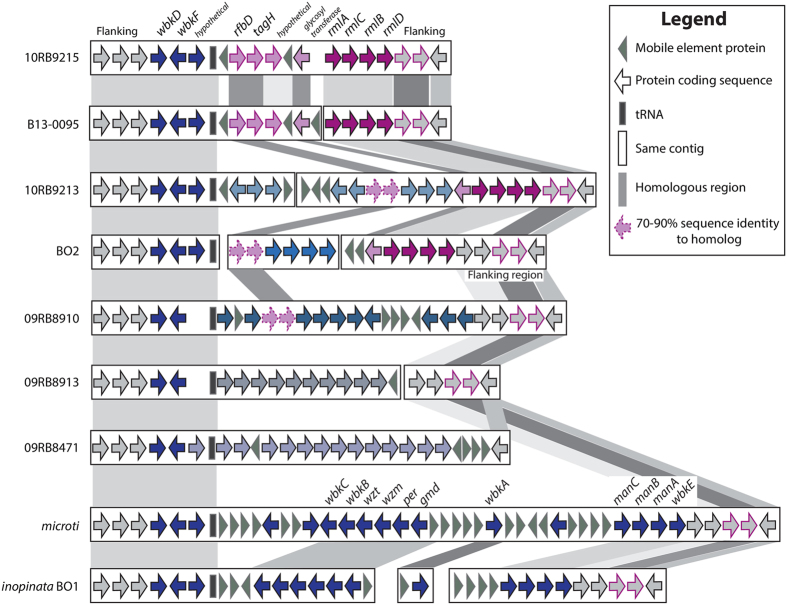
Variable organization of the *wbk* region in the *Brucella* sp. strains isolated from African bullfrogs. Comparison of the *wbk* region, involved in the synthesis of LPS in the classical *Brucella* strains, across *B. microti, B. inopinata* (BO1), the BO2 strain, B13-0095 isolated from a Pacman frog and five African bullfrog strains. Genes that are shared across different species have similar color and border patterns, and are also indicated by background shading. Genes with sequence similarity above 91% have solid borders and shading, but those with similarity between 70–90% are indicated by dashed borders. Coding sequences appear as arrows, mobile element proteins as grey triangles and tRNAs as solid boxes. Genes united on the same contig or chromosome are found within the same rectangle. The classically known *wbk* region can be seen in *B. microti* and *B. inopinata*. All genes, their product description, the assigned protein family, and the gene order in the newly annotated genomes are identified by a peg identifier (Supplementary Table S3).

As each bullfrog strain had a unique S-LPS profile, we specifically searched for the nineteen genes in the *wbk* region that are essential for LPS synthesis^26^,^27^, the novel genes (*rmlACBD*) found in the BO2^28^ and B13-0095^22^ isolates, as well as the flanking areas of those regions. All *Brucella* genomes share the same 5′ flanking region (Fig. 5), and each of the genomes described here has a unique organization beyond the conserved 5′ end of *wbkF*, although 10RB9215 is nearly identical to the previously described B13-0095 genome (Fig. 5 and Supplementary Table S3). None of the frog isolates had a profile similar to that seen in the classical *Brucella* spp. and *B. inopinata* (BO1) genomes or to the novel configuration that strain BO2 has.

The *rmlACBD* region is shared across two of the bullfrog strains (10RB9213 and 10R9215) as well as the previously described BO2 and B13-0095. Strains 10RB9215 and B13-0095 also share four genes upstream of this region. Two of these (*rfbD* and *tagH*) have been previously noted in the B13-0095 and BO2^22^ genomes and are also found in strain 09RB8910. All proteins were the best bidirectional BLASTP hits to each other, but while the sequence similarity was greater than 99%, these two genes have somewhat reduced sequence similarity in BO2, 10RB9213 and 09RB8910. Another gene in this area, annotated as a glycosyl transferase, is also shared across these same genomes except for 09RB8910 (Fig. 5).

The *wbk* region is strikingly similar between *B. microti* and *B. inopinata* (BO1), as well as highly similar in strains 10RB9215 and B13-0095. In contrast, the bullfrog isolates and BO2 have unique genes in this region (Fig. 5 and Supplementary Table S3).

Two other genes, *wboA* and *wboB*, known to be important in LPS synthesis, are located outside of the *wbk* region. The *wboAB* genes^27^ are missing in all bullfrog strains, strain B13-0095, and strain BO2, but are present in *B. inopinata* (BO1) and all of the classical species.

#### Flagellar genes

**Figure 6:**
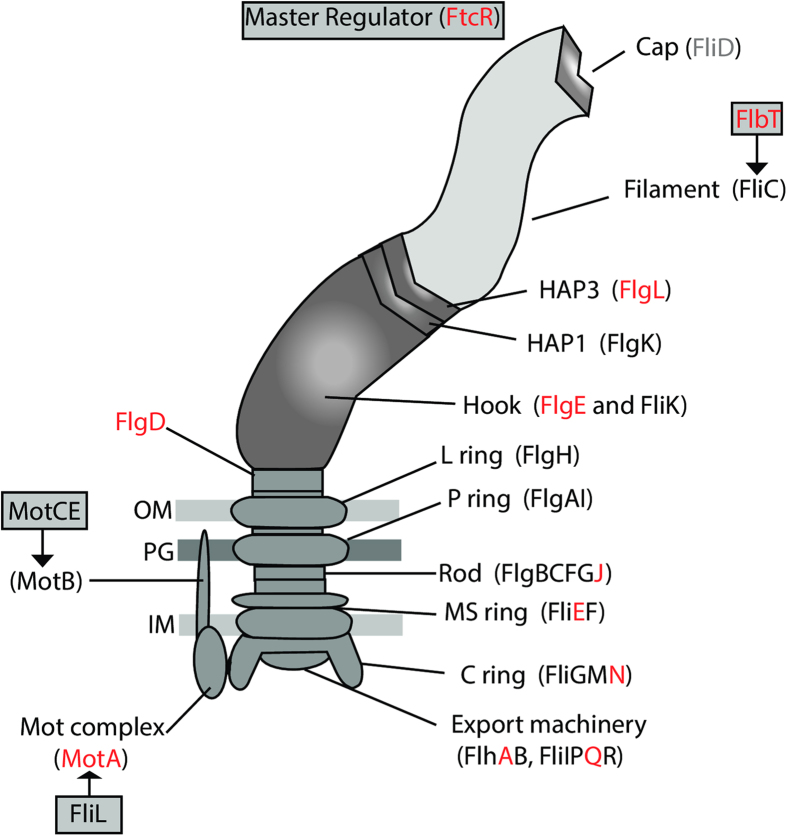
Schematic representation of flagellar gene expression. Regulators of flagellar gene expression and proteins participating in assembly of the flagellum. Those genes that appear to be functional across all of the compared strains (see Supplementary Table S4) are marked in red.

An initial examination of the genes from the African bullfrog strains, the BO1 and BO2 strains, and *B. microti* CCM 4915 revealed an interesting pattern of pseudogenization, loss, or in-frame deletions of many of the flagellar genes. The examination was expanded to look across the major species and strains of *Brucella* as previously described^11^. A summary of the presence, absence, or sequence disparities among the known flagellar genes^29^,^30^,^31^ is provided (Supplementary Table S4). Figure 6 shows the genes involved in the construction and regulation of the flagellum, with red lettering indicating those few genes that appear to be functional in all of the compared strains.

Most of the classical *Brucella* examined had pseudogenized at least one, if not many of the flagellar genes. *Brucella microti* was the only exception, having no pseudogenes, but it did have two genes with internal deletions (*fliG* and *flgI*) and turned out to be immotile. There is no pattern of pseudogenization in the other species of the classical clade, but instead loss of function of different flagella genes appears in each species. Within the species, some strains revealed pseudogenes that appear to be functional in their close relatives (Supplementary Table S4). Fewer pseudogenes are seen in strain BO2 and in the isolates from Australian rodents, and all genes in strain BO1 appear to be fully functional.

The flagella genes from the five African bullfrog strains we examined appear to be mutation-free. The only exceptions are in 10RB9213′s *motE* that has an internal deletion of 5aa, and 09RB8910′s *flgI* gene that has an upstream mutation which shortens it to 409aa, a mutation shared with the genomes in the ancestral clade (Supplementary Table S4). As all bullfrog strains are motile, these few mutations apparently do not influence motility.

### Infection experiments

The pathogenic potential of *Brucella* spp. correlates with their capacity to replicate in host macrophage cells and in target organs. The fate of the amphibian *Brucella* strains in mammalian macrophages and in the murine model of infection is of particular interest because African bullfrogs have been described as the first cold-blooded host of *Brucella*.

#### J774 macrophage infection

**Figure 7:**
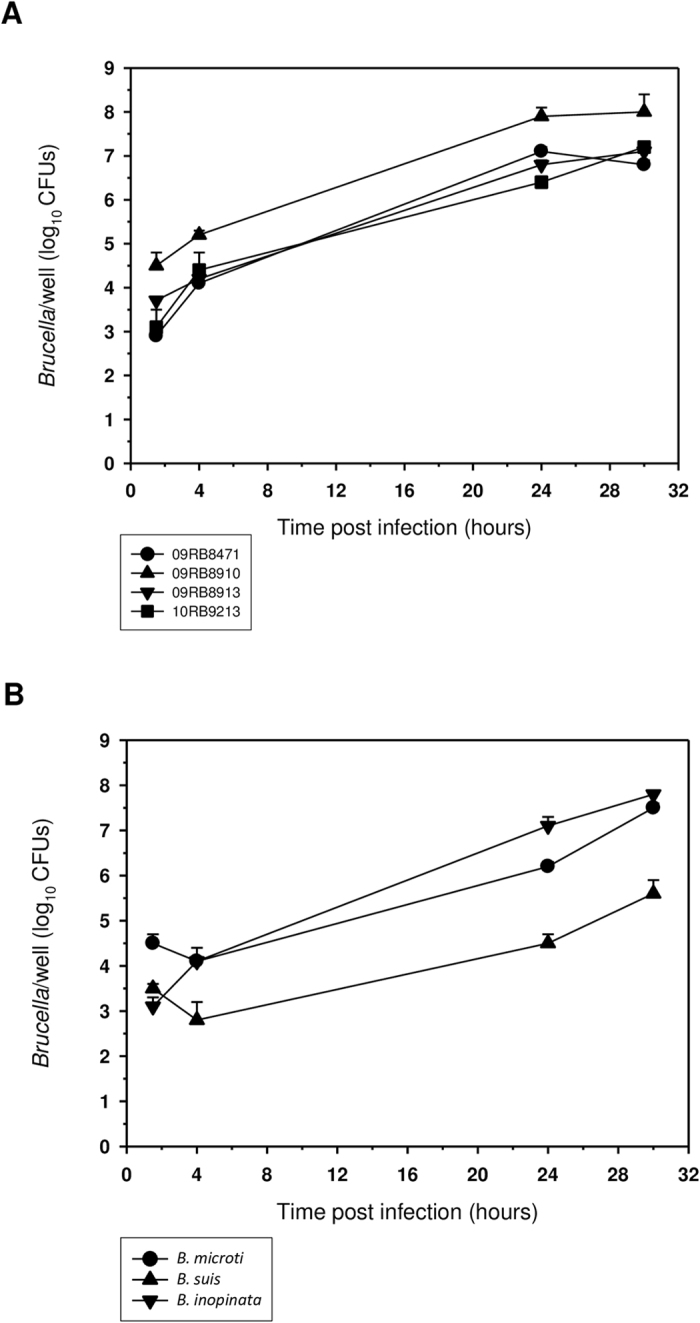
Intracellular replication of the amphibian *Brucella* sp. strains in macrophages. Intracellular multiplication of (**A**) the African bullfrog strains 09RB8471 (●), 09RB8910 (▲), 09RB8913 (▼) and 10RB9213 (■) in comparison with (**B**) *B. microti* CCM 4915 (●), *B. inopinata* BO1 (▼), and *B. suis* 1330 (▲) in murine J774 macrophage-like cells. All experiments were performed three times in triplicate each, and results of one typical experiment are shown, presented as the means ± standard deviation.

Brucellae isolated from amphibians readily multiplied in J774 murine macrophage-like cells (Fig. 7A). However, there were significant differences between the ability of our amphibian strains and classical *B. suis* 1330 to replicate within mammalian macrophages. At 24 h post-infection, the number of intracellular bacteria was ~2 to 4 logs higher than that of *B. suis* 1330 (Student’s t-test, p ≤ 0.01). The observed reduction in viable intracellular counts for at least one out of four bullfrog strains (09RB8471) at 30 h post-infection may be explained by very rapid growth, resulting in macrophage lysis and killing of released bacteria in the gentamicin-containing medium. In contrast, strain 09RB8910 reached higher bacterial loads, maybe because the infected macrophages were more efficiently protected from apoptosis^32^. Over a period of 24 h, the overall intramacrophagic growth rate of these strains was comparable to that of *B. inopinata* (BO1), and slightly higher than that of *B. microti* CCM 4915 (Fig. 7B).

#### BALB/c mice infection

The ability of the bullfrog strains to replicate in murine macrophages prompted us to analyse the susceptibility of BALB/c mice. In a preliminary experiment, 10^5^ CFU of strains 09RB8471 and 09RB8910 were intraperitoneally injected. This dose is widely used in standard murine infections with *B. suis* 1330 but is known to be lethal for BALB/c mice which are infected with *B. microti* CCM 4915, *B. inopinata* BO1, and the Australian rodent strain 83-210^33^,^34^. However, our amphibian isolates were not lethal for BALB/c mice using this specific dose.

**Figure 8:**
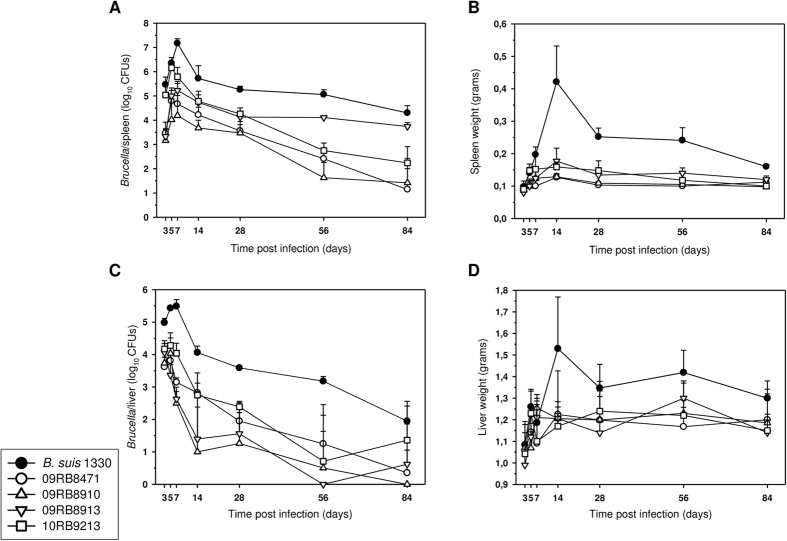
Course of amphibian *Brucella* sp. infection in BALB/c mice. Bacterial counts and organ weights of spleen and liver. Growth curves of *Brucella* sp. strains 09RB8471 (○), 09RB8910 (△), 09RB8913 (▽), 10RB9213 (□), and *B. suis* 1330 (●) in spleens (**A**) and livers (**C**) of BALB/c mice after intraperitoneal inoculation of 10^4^ colony-forming units (CFU) of the bacteria. For each infection experiment, five mice were killed per time point after 3, 5, 7, 14, 28, 56, and 84 days and the number of viable bacteria was counted. Spleen (**B**) and liver weights (**D**) were also determined. Data are presented as mean values ± standard deviation.

The pathogenic potential of the *Brucella* sp. strains isolated from African bullfrogs was also assessed by following up splenic and hepatic colonization (Fig. 8A,C) as well as spleen and liver weights (Fig. 8B,D) from day 3 to 84 post infection with 10^4^ CFU. The bacterial replication rates in spleen and liver peaked between the third and seventh day after infection (Fig. 8A,C), similar to other *Brucella* species^35^ including *B. suis* 1330. The bullfrog strains were eliminated much more rapidly from spleen and liver than *B. suis* 1330 but still persisted 84 days post infection (except for 09RB8910 in the liver, which was totally cleared by this time point). Remarkably, at the end of the experiment (84 d post infection) strain 09RB8913 showed persistence in the spleen comparable to *B. suis* 1330.

In the course of infection, the colonization rate of the target organs was significantly different between BALB/c mice infected with *B. suis* 1330 and those infected with the bullfrog strains, except for 10RB9213 in the spleen three and five days post infection, and for 10RB9213 and 09RB8913 in the liver 12 weeks post infection.

Neither spleen nor liver weight significantly increased due to the infection with the amphibian *Brucella* sp. strains indicating a very mild inflammatory reaction.

## Discussion

The bullfrog strains genetically group together, although showing considerable heterogeneity, and are basal to the core *Brucella* species (Figs 2 and 3). This amphibian group is distantly related to *Ochrobactrum* but nested within existing atypical *Brucella* species, including isolates from Australian rodents, *B. inopinata* BO1, and *Brucella* sp. strain BO2. Although it is likely that more *Brucella* species from the classic clade – species that appear to be host associated – will be discovered, new species within these new basal lineages and their genetic diversity challenge our concepts of what defines *Brucella*.

Greater genetic diversity exists among the relatively few members of the basal *Brucella* clade than in the hundreds of strains in the classical clade. This diversity may come from the ability of these basal species to exchange DNA with each other and with other microbes in the environment using horizontal gene transfer. A similar situation is seen in the *Mycobacterium* complex where *M. canettii*, an opportunistic pathogen for humans, undergoes horizontal exchanges with other mycobacteria, while *M. tuberculosis*, the host adapted obligatory pathogen, is strictly clonal^36^.

Our analysis expands the previously described ancestral clade of *Brucella*^11^ which included the isolates from Australian rodents (83/13 and NF 2653) and two novel isolates from human infections (*B. inopinata* BO1^16^ and *Brucella* sp. BO2^17^). Figure 3 shows that the basal clade of atypical strains can be divided into two subclades; the first contains the Australian rodent isolates and five of the bullfrog strains (10RB9215, 10RB9213, 10RB9214, 10RB9212, 09RB8910). The other major subclade contains *B. inopinata* (BO1), BO2, and the Pacman frog strain B13-0095, as well as four of our bullfrog strains 09RB8918, 09RB8913, 10RB9210, 09RB8471. As part of these two subclades, our bullfrog strains thus span much of the known diversity within the atypical *Brucella* species. Interestingly, *B. vulpis* is related but is basal to all of these other atypical species. Combined, these results suggest substantial undiscovered diversity in the genus.

Comparative whole genome analysis revealed a set of genes present on the chromosomes of the African bullfrog strains that are not found in other *Brucella* species but found in soil bacteria such as *Rhizobium* and *Agrobacterium (data not shown*). In contrast to their soil- and plant-associated relatives, which harbour virulence factors such as plasmids and temperate phages necessary to survive in fast changing harsh environmental conditions, having these type of genes seems to be superfluous for an organism adapted to intracellular survival^37^. Recently, we described a temperate phage residing in the human isolate *B. inopinata* that was very similar to a temperate phage of *O. anthropi* and to several prophages identified in rhizobiales^38^. These findings and the detection of pili-like structures in the *Brucella* sp. isolated from African bullfrogs may hint that horizontal gene transfer does occur between *Brucella* and other bacteria in the same niche, such as soil associated bacteria.

This notion that the basal species more readily exchange DNA with each other and other microbes is strengthened by the great diversity of new genomic regions found in the five strains more deeply analyzed, and is also apparent in the heterogeneity of the *wbk* region (Fig. 5 and Supplementary Table S3). Across the classical clade, the composition and order of *wbk* is conserved, with the only mutations seen in some of the genes from those species known to have a rough phenotype. When BO2 was first described, phenotypic analysis and sequencing showed that it had a different structure in its *wbk* region that included four new genes predicted to be involved in constructing a rhamnose-based O-antigen, making it unique at that time among *Brucella*^28^ but well described in other bacteria^39^,^40^. These genes, *rmlACBD*, encode a rhamnose-based O-antigen^28^ that is different from the N-formyl-perosamine-based O-antigen produced by classic *Brucella*^41^. As our knowledge of *Brucella* expands with the amphibian isolates, our understanding of the *wbk* region is rapidly evolving and suggests that this is an active region of change within these genomes. Strains 10RB9213 and 10RB9215 both have those same *rml* genes, but have radically different genes in the upstream flanking region. In fact, each of the amphibian representatives from this study has a unique set of genes in the *wbk* region (Fig. 5 and Supplementary Table S3), although they share (for the most part) the flanking regions at either end. They each have a variety of genes that are unique not only among strains that have been described previously, but also unique compared to each other. The sole exception is the African bullfrog strain 10RB9215 and the Pacman frog isolate B13-0095, which are almost identical in this region. It appears that horizontal transfer is a major diversifying force in this area, but it is impossible to say just what gene structures the ancestor to all the *Brucella* originally had between the conserved flanking regions. It is also impossible to discern what type of O-antigen that ancestor possessed. Comparison to *Ochrobactrum* genomes show that they have the *rmlACBD* operon, too, but here the same flanking regions are widely dispersed, with the 5′ and 3′ ends on separate chromosomes (as per *O. anthropi* ATCC 49188, *data not shown*).

The flagellar system, which is phylogenetically related to the type III secretion system and as such may be involved in the secretion of virulence factors, appears to be essential for the infectious cycle and persistence of *Brucella* in mammalian hosts. *Brucella melitensis* has been shown to produce a sheathed flagellum during the early stages of exponential growth^29^,^42^, and while *B. melitensis* with mutated flagellar genes were not attenuated in cellular models of infection, BALB/c mice were able to clear them within 12 weeks of infection^29^. When comparisons are made across all *Brucella* and the sequences of the genes that produce the flagellum, an extensive pattern of pseudogenization is apparent (Fig. 6 and Supplementary Table S4), suggesting that most, if not all of the known classical species are not motile. In contrast, the African bullfrog strains have a flagellum (Fig. 1) and are motile (Supplementary Fig. S2). BO2 also has a full component of functional genes, and motility has recently been demonstrated in this strain^22^. One could hypothesize that the strains in the classical clade, with the possible exception of *B. microti,* accumulated mutations in their flagellar genes in a random manner because they no longer require mobility. Their predominantly intracellular lifestyle may not require the presence of a flagellum.

Although primarily isolated from a cold-blooded host, the African bullfrog strains revealed long-term survival in mammals comparable to the classic *Brucella* species in BALB/c mice (up to 84 d after inoculation). In contrast to the hepatosplenomegaly observed after *B. suis* infection, intraperitoneal infection of BALB/c mice with the African bullfrog strains proceeded in the form of asymptomatic persistence. This might be explained by differences in the expression of flagellin, which is known to play a key role in the immunological stand-off between *Brucella* and its host^43^. Both the downregulation of flagellin expression as well as the changes in the amino acid sequence of flagellin may help to avoid recognition by the innate immune system of the host^43^. Whether the variable flagellar genes of the amphibian *Brucella* sp. strains may contribute to the stealthy strategy of this pathogen remains unknown. Interestingly, motility of the flagellum necessary to cope with environmental stress and the flagellum as a virulence factor for persistent infection in a mammalian host do exist in parallel in the bullfrog isolates.

A major aspect of *Brucella* virulence is the capacity of the bacteria to replicate inside macrophages and their ability to escape the host immune system. We tested the pathogenic potential of the novel amphibian isolates in both *in vitro* and *in vivo* infection models. The African bullfrog strains showed an enhanced capacity to replicate in macrophages at a level comparable to *B. microti, B. inopinata*, and the Australian rodent isolates^33^,^34^ and inconsistent with classical *B. suis*. The high replication rate in phagocytic cells correlates well with the fast growth of the bullfrog strains in culture medium. Specific metabolic pathways might be responsible for the faster growth and metabolism of the bullfrog isolates differed essentially from other brucellae (Supplementary Table S1 and Supplementary Fig. S1). However, despite the higher replication rate of these atypical *Brucella* strains *in vitro*, they apparently do not cause death in the murine model of infection. New virulence factors of the amphibian *Brucella* sp. strains such as the functional flagellum or a novel lipopolysaccharide might be responsible for their long-term persistence in mammals but further experiments are needed to address this hypothesis.

Despite their close relationship to the atypical fast-growing brucellae, the African bullfrog strains revealed long-term survival in mammalian hosts comparable to strains from the classical clade. Although the pathogenicity of brucellae *in vivo* usually correlates with their ability to replicate in macrophages, the bullfrog strains neither killed BALB/c mice with a standard infectious dose of 10^5^ bacteria such as other atypical brucellae nor caused a systemic inflammatory response associated with an increase in liver and spleen weights as seen in classical brucellae. Hence, according to our experimental data and considering the potential misidentification as *Ochrobactrum* sp. as well as the difficulties in serological tests due to modifications of the LPS, the potential infections of livestock and humans by the amphibian *Brucella* sp. cannot be easily assessed.

## Concluding Remarks

In the last decade, the emergence of several new *Brucella* species as well as atypical *Brucella* strains have enhanced our understanding of the evolution of the genus from a soil-associated motile bacterium to a host adapted clonal pathogen. Our results greatly extend understanding of the genomic and phenotypic diversity of atypical *Brucella*. The amphibian strains that are described here form a remarkable group of heterogeneous brucellae, characterized by some unique features. First, *Brucella*-like organisms have been described from African bullfrogs and other frogs, but this is the first comprehensive description of variable *Brucella* genomes from a single cold-blooded host. Interestingly, the *Brucella* sp. strains isolated from African bullfrogs revealed versatile adaptability both to cold-blooded animals and endotherms. Second, while a single amphibian strain was recently shown to be motile, our studies confirm that motility is a common feature of diverse organisms that are progenitors to the classical zoonotic *Brucella*. Third, the bullfrog strains show long-term persistence in BALB/c mice without causing disease. The zoonotic potential and pathogenicity of these *Brucella* sp. strains must be assessed carefully because of their close phylogenetic relationship to the human opportunistic pathogens *B. inopinata* (BO1) and *Brucella* sp. strain BO2.

Our data support the hypothesis that the classical core *Brucella* members have emerged from soil bacteria that are characterized by motility and a broad metabolic activity. *Brucella* evolved to become a highly virulent but host specific clonal pathogen by genome reduction and adaptation. However, the apparent competence of the amphibian strains to exchange DNA may allow *Brucella* to adapt to changing environments quickly and give it a broad range of hosts.

## Methods

### Bacterial strains

A total of 21 strains (Table 1) isolated from tissue samples of various moribund or dead African bullfrogs kept in a quarantine unit of the Frankfurt Zoo (Germany) were analyzed^44^. The animals had been originally imported from Tanzania.

The bacteria were grown on *Brucella* agar and Columbia agar for 48 h at 37 °C both with and without 10% CO_2_. Molecular analyses (16S rRNA [*rrs*], *recA*, multilocus sequence typing [MLST], and multiple-locus variable-number tandem repeat analysis [MLVA]) as well as phenotypic characterization (biochemical profiling, agglutination, phage lysis) were performed as described previously for *B. microti* isolates^10^. Whole genome sequencing was conducted on nine representative strains.

### Phenotyping

Classical microbiological methods were employed, that determine CO_2_ requirement, H_2_S production, urea hydrolysis, agglutination with monospecific sera (anti-A, anti-M, and anti-R), dye sensitivity (basic fuchsin and thionine), and phage lysis (F1, F25, Tb, BK2, Iz, Wb, R/C) for the identification and sub-differentiation of the African bullfrog strains^45^.

Since members of the atypical group of novel emerging *Brucella* species display lipopolysaccharide (LPS) heterogeneity^25^,^28^, structural differences in LPS were investigated using monoclonal antibodies (mAbs) specific for O-polysaccharide (O-PS) epitopes: 2E11 (IgG3; M epitope), 12G12 (IgG1; C [A = M] epitope), 12B12 (IgG3; C [M > A] epitope), 18H08 (IgA; C/Y [A = M] epitope), 05D4 (IgG1; C/Y [A > M] epitope). An ELISA was set up with hybridoma supernatants including mAbs and whole-cell bacterial antigens. SDS-PAGE of proteinase K-digested S-LPS preparations followed either by silver staining or Western blotting was performed as described previously^25^.

Metabolic activity was evaluated using a commercial biotyping system (Micronaut; Merlin Diagnostika, Bornheim-Hersel, Germany) as described previously^23^. The 96-well Micronaut^®^ BfR *Brucella* assay tests for 29 aminopeptidases, two phosphatases, four glucosidases, one esterase, and the metabolism of eleven monosaccharides, three disaccharides, seven sugar derivates, 15 amino acids, eleven organic acids, one salt, one amino acid derivate, one peptide, one base, and six classical reactions (nitrite and nitrate reduction, pyrazinamidase, Voges-Proskauer medium, urease, and H_2_S production). Biotyping experiments were carried out in triplicates. Hierarchical cluster analysis was conducted by Ward’s linkage algorithm using binary coded data based on empirically set cutoffs (Bionumerics v. 7.5, Applied Maths, Sint-Martens-Latem, Belgium). All characters were considered equal within the data set.

Motility of the bacteria was tested using a 0.3% semisolid agar including TTC (2,3,5 triphenyltetrazolium chloride) to visualize dynamics of bacterial swarming. Exemplarily, *Brucella* sp. strain 10RB9206 was harvested after 12, 24, 36, 48, 60, 72, and 84 h of culture to analyze the ultrastructure of a prospective flagellum using transmission electron microscopy (JEM-1010 electron microscope). Bacterial samples were prepared for microscopic examination by negative staining with 1% solution of uranyl acetate dissolved in distilled water (pH 4.2 to 4.5).

### DNA preparations

A single colony of each strain was transferred from the agar plate to 200 μl 5x lysis buffer D (PCR Optimizer kit; Invitrogen, De Schelp, The Netherlands) diluted 1:5 in distilled water, supplemented with 0.5% Tween 20 (ICI America Inc., Merck, Hohenbrunn, Germany) and 2 mg/ml proteinase K (Roche Diagnostics, Mannheim, Germany). The bacterial samples were incubated at 56 °C for 1 h and inactivated at 95 °C for 10 min, before crude DNA samples were purified using the QIAamp DNA Mini kit (Qiagen, Hilden, Germany) according to manufacturer’s instructions.

High quality genomic DNA (gDNA) was prepared for whole genome sequencing by using a Qiagen genomic extraction kit and Qiagen Genomic-tip 100/G (Qiagen, Hilden, Germany) according to manufacturer’s recommendations. Bacterial lysis was optimized by three freeze/thaw cycles in a lysozyme/lysostaphin buffer.

### IS*711, bcsp31*, and Bruce-ladder multiplex PCR

The presence of both the *Brucella*-specific *bscp31* gene and insertion element IS*711* was determined^19^ and the Bruce-ladder multiplex PCR was used in its modified version^46^. Presence of IS*711* and its copy number were also assessed by Southern blot analysis as described previously^47^.

### Analysis of 16S rRNA and *recA* genes

16S rRNA (*rrs*) and *recA* (recombinase A) genes were amplified and sequenced as described previously^48^,^49^. Almost the entire *rrs* sequence was amplified using universal primers, i.e. 27f and 1492r (all primer sequences can be retrieved from Al Dahouk *et al*.)^10^. The primer pair *recA*-BrucOchro-f and *recA*-BrucOchro-r generated a fragment including the whole *recA* gene (1,086 bp). 15 pmol of each primer were added to 50 μl Ready-To-Go master mix (Eppendorf GmbH, Hamburg, Germany) and PCRs were carried out in a Perkin-Elmer GeneAmp 2400 thermal cycler (Perkin-Elmer, Applied Biosystems, Foster City, CA, USA). A total of 30 cycles were performed: 30 s of denaturation at 94 °C, 30 s of annealing at 58 °C (*rrs*) or 65 °C (*recA*), and elongation at 72 °C for 90 s (*rrs*) or 60 s (*recA*). The run was completed with a final elongation step of 7 min at 72 °C. PCR products were analyzed by agarose gel electrophoresis (1% [wt/vol] in Tris-Acetate-EDTA [TAE] buffer). The purified *recA* and *rrs* fragments were sequenced with an ABI Prism 3100 genetic analyzer (Applied Biosystems, Foster City, CA, USA) using *recA*-BrucOchro-f/*recA*-BrucOchro-r primers and an internal primer set consisting of 341fw, 518r, and 926 f, respectively. ClustalW, version 1.8, was used (http://clustalw.genome.jp/) to generate multiple sequences alignments.

### MLSA and MLVA

Multilocus sequence analysis was performed as described previously^50^ by determining DNA sequences at nine independent genetic loci. In order to establish the relationships of the amphibian isolates, these sequence data were compared with equivalent data from the 27 sequence types (STs) originally described, representing all *Brucella* species and biovars recognized at the time as well as more recently emerged *Brucella* spp. including the type strains of *B. microti, B. papionis, B. inopinata*, an organism described as *B. inopinata*-like (strain BO2)^17^ and a representative of a group of atypical isolates from Australian rodents^18^. Sequences for eight of the nine loci examined (excluding one not present in the *Ochrobactrum* outgroup) were concatenated as described previously^19^,^21^. Alignment and phylogenetic analysis were performed within the MEGA 5.2 package using the Jukes-Cantor distance and the neighbor joining approach.

Multiple-locus variable-number tandem repeat (VNTR) analysis (MLVA-16) was carried out as described by Le Flèche and colleagues^51^ and modified by Al Dahouk and colleagues^52^ using eight minisatellite markers (*panel 1*: bruce06, bruce08, bruce11, bruce12, bruce42, bruce43, bruce45, bruce55) and eight microsatellite markers (*panel 2A*: bruce18, bruce19, bruce21; panel 2B: bruce04, bruce07, bruce09, bruce16, bruce30). Alleles were called according to version 3.6 of the *Brucella* table for allele assignment (MLVA allele-coding convention to convert allele size (bp) into number of repeats (u)).

### Phylogenetic analysis

Comparative genomic analyses were conducted following previously described methodology^53^,^54^ to discover single nucleotide polymorphisms (SNPs). Representative strains clustering in different groups were selected according to molecular typing data. Genome sequencing was carried out by the sequencing services provider GATC Biotech, Konstanz, Germany using either MiSeq (Illumina Inc., San Diego, CA, USA) or PacBio (Pacific Biosciences, Menlo Park, CA, USA). De novo assembly of the Illumina and PacBio sequencing reads was done with CLC Genomics Workbench 9.0 (CLC bio, Aarhus, Denmark) and SMRT Analysis Software v. 2.3 (Pacific Biosciences), respectively. Illumina reads were assembled using standard parameters. For the assembly of PacBio reads, the HGAP3 algorithm with a read length minimum of 2,500 bp was used. SNP analysis was conducted with the NASP v. 1.0.0 pipeline (https://github.com/TGenNorth/NASP) using default settings for the programs implemented in the pipeline. Briefly, assemblies of the amphibian *Brucella* sp. in FASTA format were aligned to the *B. abortus* 2308 reference genome (GenBank accession numbers NC_007618, NC_007624) and analyzed for SNPs with MUMmer 3.23^55^; SNP discovery using sequence reads gave nearly identical results (*data not shown*). Reads were aligned to the reference with BWA v. 0.3.7^56^, and SNPs discovered in the alignments with the Genome Analysis Toolkit v. 2.5 Unified Genotyper^57^. We required SNP loci had have a base call in all samples, i.e., no missing data. SNP loci in duplicated regions, determined by an alignment of the reference to itself with MUMmer, were excluded from analyses. Phylogenetic trees were built using maximum parsimony in PAUP* with 100 bootstrap replicates to indicate the amount of support for various branches^58^. Trees were visualized with FigTree v. 1.4.0 (http://tree.bio.ed.ac.uk/software/figtree/). Eight of the classical *Brucella* species were included in the tree for phylogenetic context and to contrast the amounts of genetic diversity among the various clades. *Ochrobactrum anthropi* ATCC 49188^T^ (GenBank accession numbers CP000758-CP000763) was used as an outgroup.

### Comparative analysis

The comparative gene-based analysis included the genomes of strains 10RB9213, 10RB9215, 09RB8910, 09RB8913, and 09RB8471. An initial examination using 16S rRNA revealed that some bullfrog isolates were closely related to *B. inopinata* BO1^19^, so these genomes were compared to *B. inopinata* BO1. *Brucella* sp. BO2 and *B. microti* CCM 4915^T^ were also chosen for the initial comparison. All genomes were annotated with RASTtk to provide consistency across the results. A list of all genomes used, as well as their annotation statistics, is provided (Table 3).

Several methodologies were used to compare the genomes, all of which are available in PATRIC^53^. The Protein Family Sorter^54^ was used to look for unique regions in individual genomes. Regions determined to be unique to each bullfrog strain, or shared across them, were verified by BLAST against the representative genome database at NCBI. In addition, these unique regions were examined by BLAST against both the complete plasmid and complete bacteriophage databases.

A detailed comparison of specific genomes (10RB9215, B13-0095, 10RB9213, BO2, 09RB8910, 09RB8913, 09RB8471, *B. microti* CCM 4917 and *B. inopinata* BO1) was conducted to analyze the *wbk* region. Contigs from each of these genomes were re-annotated using the current version of RASTtk^59^ available in the PATRIC bioinformatics resource^53^,^60^. Annotation in PATRIC now assigns protein families that are scoped at the genus level, and these PLFams were compared for this analysis^61^. The annotated genomes were first compared in a bi-directional, best BLASTP hit analysis using PATRIC’s Proteome Comparison tool^60^. The *wbk* region was selected from genome, with special attention paid to the flanking genes and the contigs they were isolated on, as well as the strength of the BLASTP hit, which included the % Sequence Identity, the % Sequence Coverage, and the directionality (uni or bi) of the hits. Only bidirectional hits were used. The PLFams were noted for all of the genes that matched these criteria. If there was a discrepancy between the PLFam assigned to each gene, a multiple sequence alignment was generated in PATRIC and examined. Following this examination, it was determined that genes with a Sequence Identity greater than 70% would be considered homologs.

A comparison of differences in metabolic pathways among the strains was made using the Comparative Pathway Tool^53^,^54^. When gene-specific differences were identified as unique to a particular genome, we looked more broadly across the different species of *Brucella* to examine consistency of results. We examined all available genomes at the species level to see if those differences were confined to the particular genome, which could indicate sequencing error, or were shared across most of the genomes available for the species.

### Macrophage infection model

The replication of brucellae within macrophages is a prerequisite for virulence of the pathogen in a given host species. Using macrophage-like murine J774-cells, we therefore compared the behaviour of *Brucella* strains isolated from bullfrogs to that of the pathogenic *B. suis* strain 1330 and of the two fast-growing species *B. microti* (strain CCM 4915) and *B. inopinata* (strain BO1). For macrophage and murine infections, all strains have been cultivated on Tryptic Soy Broth (Biokar Diagnostics, Allonne, France) for 48–72 h.

Based on molecular and biotyping data, 4 of the most diverse of the 21 bullfrog isolates were selected for *in vitro* infection experiments; namely strains 09RB8471, 09RB8910, 09RB8913, and 10RB9213. Infection experiments were performed as described previously^33^. Briefly, adherent cells were resuspended at 2.5 × 10^5^ cells/ml in RPMI 1640 cell culture medium supplemented with 10% foetal calf serum (FCS), and incubated for 24 h at 37 °C with 5% CO_2_ prior to infection. The cells were infected at a multiplicity of infection (MOI) of 20 with early-stationary phase bacteria cultured in TS medium. After 30 min, cells were washed twice with phosphate-buffered saline (PBS) and incubated in RPMI 1640/10% FCS with gentamicin (30 μg/ml) for at least 1 h to kill non-phagocytosed bacteria. Ninety minutes, 4, 24, and 30 h post-infection, cells were washed twice with PBS and lysed in 0.2% Triton X-100. CFUs were determined by plating serial dilutions on TS agar, followed by incubation at 37 °C for 2–3 days. All experiments were performed twice in triplicate.

### Murine infection model

The mouse experiments were approved by the ethical review committee of the Centro de Investigación y Tecnología Agroalimentaria, Unidad de Sanidad Animal, Zaragoza, Spain (approval no. I95/2010–1 and I111/2010-1). Well-established experimentation guidelines were followed^33^,^34^. Eight weeks old, female BALB/c mice were provided by Charles River Laboratories (Chatillon-sur-Chalaronne, France). The four bullfrog isolates chosen for the macrophage infection experiments and *B. suis* 1330 as a control were intraperitoneally injected into the mice using 10^4^ CFU in a single dose. Five animals per group were euthanized by CO_2_ asphyxiation after 3, 5, 7, 14, 28, 56, and 84 days. Spleens and livers were aseptically removed, weighed, homogenized, serially diluted in PBS, and plated onto Blood Agar Base (BAB) plates to count bacteria. Student’s t-test was applied to test for significant differences between the groups in the course of time, with *p* values ≤ 0.05 considered significant.

## Additional Information

**How to cite this article**: Al Dahouk, S. *et al. Brucella* spp. of amphibians comprise genomically diverse motile strains competent for replication in macrophages and survival in mammalian hosts. *Sci. Rep.* **7**, 44420; doi: 10.1038/srep44420 (2017).

**Publisher's note:** Springer Nature remains neutral with regard to jurisdictional claims in published maps and institutional affiliations.

## Supplementary Material

Supplementary Information

## Figures and Tables

**Table 1:** Classical phenotypic characterization of the bullfrog isolates (*strains 09RB8471 and 10RB9215 were first described by Eisenberg and colleagues^19^).

Strain no.	African bullfrog (animal no.)	CO_2_ requirement	H_2_S production	urea hydrolysis [min]	agglutination with			growth on		phage lysis using
					anti-A	anti-M	anti-R	fuchsin	thionine	F1, F25, Tb, BK2, Iz, Wb, R/C
09RB8471*	1	−	+	10	−	−	−	+	+	NL
10RB9206	1	−	+	10	−	−	−	+	+	NL
09RB8908	2	−	+	25	−	−	−	+	+	NL
09RB8909	2	−	+	25	−	−	−	+	+	NL
09RB8910	2	−	+	25	−	−	−	+	+	NL
09RB8913	3	−	+	10	−	−	−	+	+	NL
09RB8914	3	−	+	10	−	−	−	+	+	NL
09RB8915	3	−	+	10	−	−	−	+	+	NL
09RB8918	3	−	(+)	10	−	−	−	+	+	NL
10RB9205	4	−	+	10	−	−	−	+	+	NL
10RB9207	5	−	+	10	−	−	−	+	+	NL
10RB9208	6	−	+	10	−	−	−	+	+	NL
10RB9209	7	−	+	10	−	−	−	+	+	NL
10RB9210	8	−	+	10	−	−	−	+	+	NL
10RB9211	9	−	+	45	1:10	−	−	+	+	NL
10RB9212	10	−	+	45	1:10	−	−	+	+	NL
10RB9213	11	−	+	90	−	−	−	+	+	NL
10RB9214	12	−	+	40	−	−	−	+	+	NL
10RB9215*	13	−	+	80	1:20	−	−	+	+	NL
10RB9216	14	−	+	10	−	−	−	+	+	NL
10RB9217	15	−	+	10	−	−	−	+	+	NL

NL = no lysis.

**Table 2:** Summary of molecular typing applied to all 21 bullfrog isolates (*no detectable Bruce11 amplification product).

Strain no.	16S rRNA type	*recA* group	MLVA8	MLVA11	MLSA
10RB9215	A (lacks 44 nt insert)	unique	105	196	A
10RB9213	A (lacks 44 nt insert)	unique	101	202	B
10RB9212	A (lacks 44 nt insert)	2	103	197	C
10RB9214	A (lacks 44 nt insert)	2	103*	198*	C
10RB9211	A (lacks 44 nt insert)	2	103	197	C
09RB8910	A (lacks 44 nt insert)	2	103	197	C
09RB8909	A (lacks 44 nt insert)	2	103	197	C
09RB8908	A (lacks 44 nt insert)	2	103	197	C
09RB8915	B (with insert)	3	102*	201*	D
09RB8918	B (with insert)	3	102*	201*	D
09RB8914	B (with insert)	3	102*	201*	D
09RB8913	B (with insert)	3	102*	201*	D
10RB9210	B (with insert)	1	104	200	E (1)
10RB9217	B (with insert)	1	104	199	E (1)
10RB9207	B (with insert)	1	104	199	E (1)
10RB9205	B (with insert)	1	104	199	E (1)
09RB8471	B (with insert)	1	104	199	E (2)
10RB9206	B (with insert)	1	104	199	E (2)
10RB9208	B (with insert)	1	104	199	E (2)
10RB9209	B (with insert)	1	104	199	E (2)
10RB9216	B (with insert)	1	104	199	E (2)

Formally assigned sequence types (STs) released on the *Brucella* MLST database (https://pubmlst.org/brucella/): A = ST67, B = ST68, C = ST64, D = ST65, E (1) = ST66, E (2) = ST63.

**Table 3:** List of all genomes included in our analyses and their annotation statistics.

*Brucella* species	Strain	Biovar	Bioproject	Contigs	Size	CDS	Host
*abortus*	2308	1	PRJNA16203	2	3278307	3467	Bovine
*melitensis*	16 M	1	PRJNA180	2	3294931	3499	Goat
*suis*	1330	1	PRJNA320	2	3315175	3432	Swine
*suis*	ATCC 23445	2	PRJNA20371	2	3324607	3591	Swine
*suis*	686	3	PRJNA33035	23	3293432	3075	Swine
*suis*	513	5	PRJNA33033	19	3322670	3122	Rodent
*canis*	ATCC 23365		PRJNA20243	2	3312769	3435	Dog
*ovis*	ATCC 25840		PRJNA12514	2	3275590	3543	Sheep
*ceti*	Cudo		PRJNA33611	7	3389269	3154	Bottlenose dolphin
*ceti*	M644/93/10		PRJNA33041	17	3333274	3435	Hooded seal
*pinnipedialis*	B2/94		PRJNA33039	19	3395677	3414	Common seal
*microti*	CCM 4915		PRJNA32233	2	3337369	3299	Common vole
*papionis*	F8/08-60		PRJNA36511	17	3292635	3531	Baboon
sp.	NF2653		PRJNA41859	113	3104330	3250	Rodent
*inopinata*	BO1		PRJNA41855	55	3366774	3362	Human
*inopinata*-like	BO2		PRJNA41857	174	3305941	3317	Human
sp.	B13-0095		PRJNA326393	37	3360799	3325	Pacman frog
sp.	10RB9213			62	3510722	3486	African bullfrog
sp.	10RB9215		ERA672254	2	3562813	3514	African bullfrog
sp.	09RB8910		PRJNA347474	2	3592978	3615	African bullfrog
sp.	09RB8913			110	3336969	3320	African bullfrog
sp.	09RB8471		PRJNA347475	2	3434749	3491	African bullfrog
