# Supplementary material for: Brucella spp. of amphibians comprise genomically diverse motile strains competent for replication in macrophages and survival in mammalian hosts
Source: Sci Rep. 2017 Mar 16;7:44420. doi: 10.1038/srep44420 (PMC5353553; doi:10.1038/srep44420)
Supplement: Supplementary Information [file srep44420-s1.pdf]

Supplementary Information for:

Brucella spp. of amphibians comprise genomically diverse motile strains competent for replication in macrophages and survival in mammalian hosts

Sascha Al Dahouk, Stephan Köhler, Alessandra Occhialini, María Pilar Jiménez de Bagüés, Jens Andre Hammerl, Tobias Eisenberg, Gilles Vergnaud, Axel Cloeckaert, Michel S. Zygmunt, Adrian M. Whatmore, Falk Melzer, Kevin P. Drees, Jeffrey T. Foster, Alice R. Wattam, Holger C. Scholz

Content:

Supplementary Figures

Supplementary Figure S1. Hierarchical cluster analysis of *Brucella* species and the African bullfrog strains based on 93 biochemical reactions tested with the *Brucella*-specific Micronaut™ microtiter plate performed by the Ward´s linkage algorithm.

Supplementary Figure S2. Motility of the amphibian isolates (09RB8471 (**A**), 09RB8910 (**B**), 09RB8913 (**C**), and 10RB9213 (**D**)), *B. melitensis* 16M (**E**), and *O. anthropi* LMG 3331 (**F**) was tested using a 0.3% semisolid agar including TTC (2,3,5 triphenyltetrazolium chloride) to visualize bacterial swarming.

Supplementary Figure S3. Condensed dendrogram of clustered MLVA-11 genotypes of *Brucella* spp. The bars reflect the percentages of divergence. The cluster of the *Brucella* sp. strains isolated from African bullfrogs and its relationship to closely related species, such as *B. inopinata*, *B. microti*, *B. ceti*, and *B. pinnipedialis* are presented in more detail.

Supplementary Figure S4. Minimum spanning tree comparing MLVA-16 data of approximately 3,500 strains from various *Brucella* species, some biovars and significant subspecies clusters in comparison with the African bullfrog strains. Data can be queried at http://microbesgenotyping.i2bc.paris-saclay.fr/, the *Brucella* MLVA cooperative database. Categorical distance was used and the creation of hypothetical intermediate links was allowed. Connecting branches are shown up to a length of 6.

Supplementary Tables

Supplementary Table S1. Metabolic activity of *Brucella* sp. isolates from African bullfrogs in comparison with classical and newly described species tested with the Micronaut® BfR *Brucella* assay (-, no metabolic activity; +, metabolic activity; v, variable metabolic activity). *Brucella*-specific traits are shown in boldface.

Supplementary Table S2. Genes unique to each of the new *Brucella* genomes, with an indication of their contig, start and stop location, gene order, contig location, DNA and amino acid size, strand, protein family, product description and an indication if they are shared across the other novel strains by black shading.

Supplementary Table S3. The genes that constitute the *wbk* region of the bullfrog isolates 10RB9215, 10RB9213, 09RB8913, 09RB8910, and 09RB8471 in comparison with the Pacman frog isolate B13-0095, *B. microti*, *B. inopinata* (BO1), and strain BO2.

Supplementary Table S4. Summary of the presence, absence, or sequence disparities among the flagellar genes of classical (*B. melitensis*, *B. abortus*, *B. suis*, *B. canis*, *B. ovis*), newly described and atypical brucellae (*B. ceti*, *B. pinnipedialis*, *B. microti*, *B. papionis*, *B. inopinata* (BO1) and strain BO2), the isolates from Australian rodents (Aus) and African bullfrogs.

Suppl. Figure S1

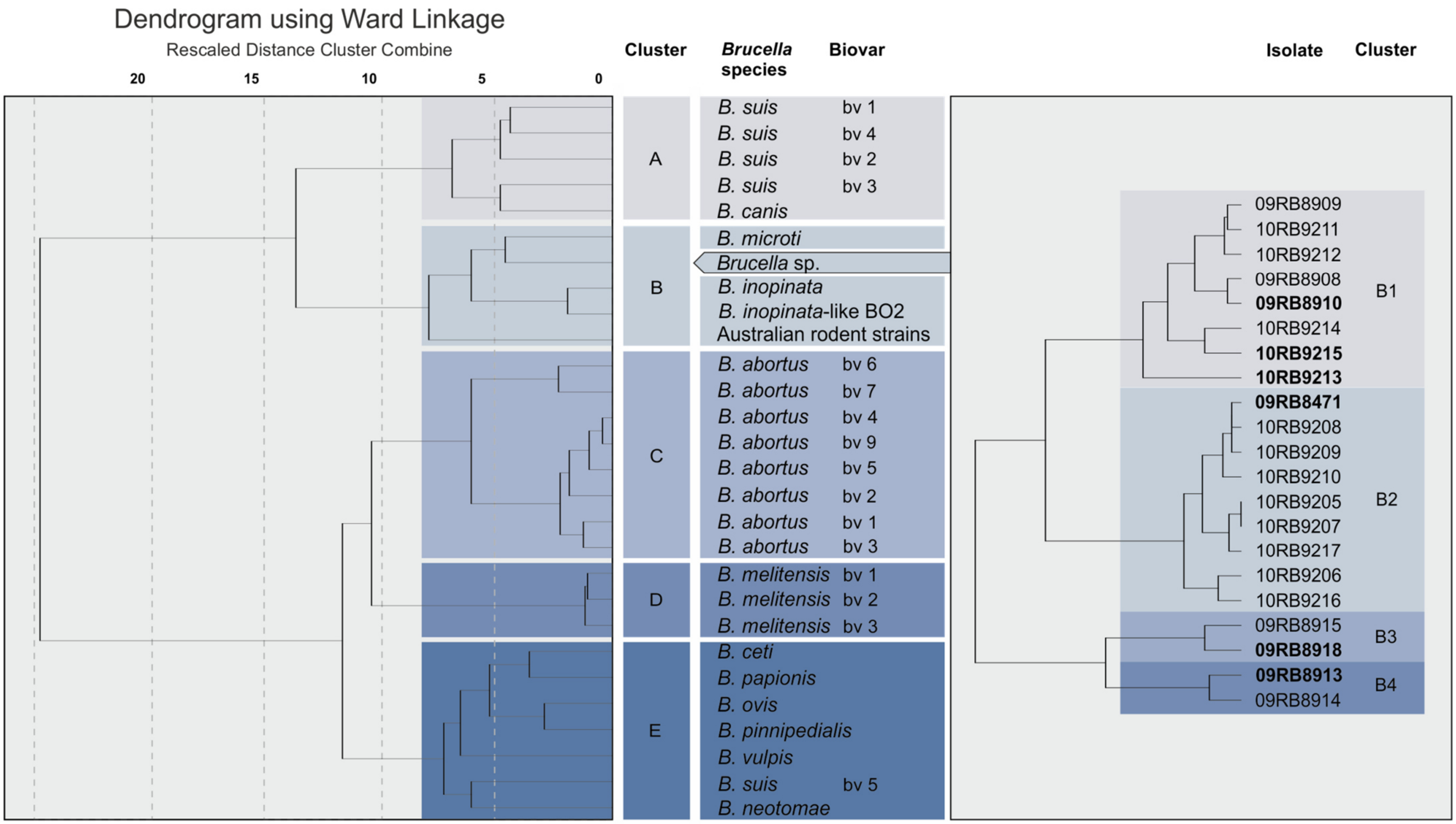

Suppl. Figure S2

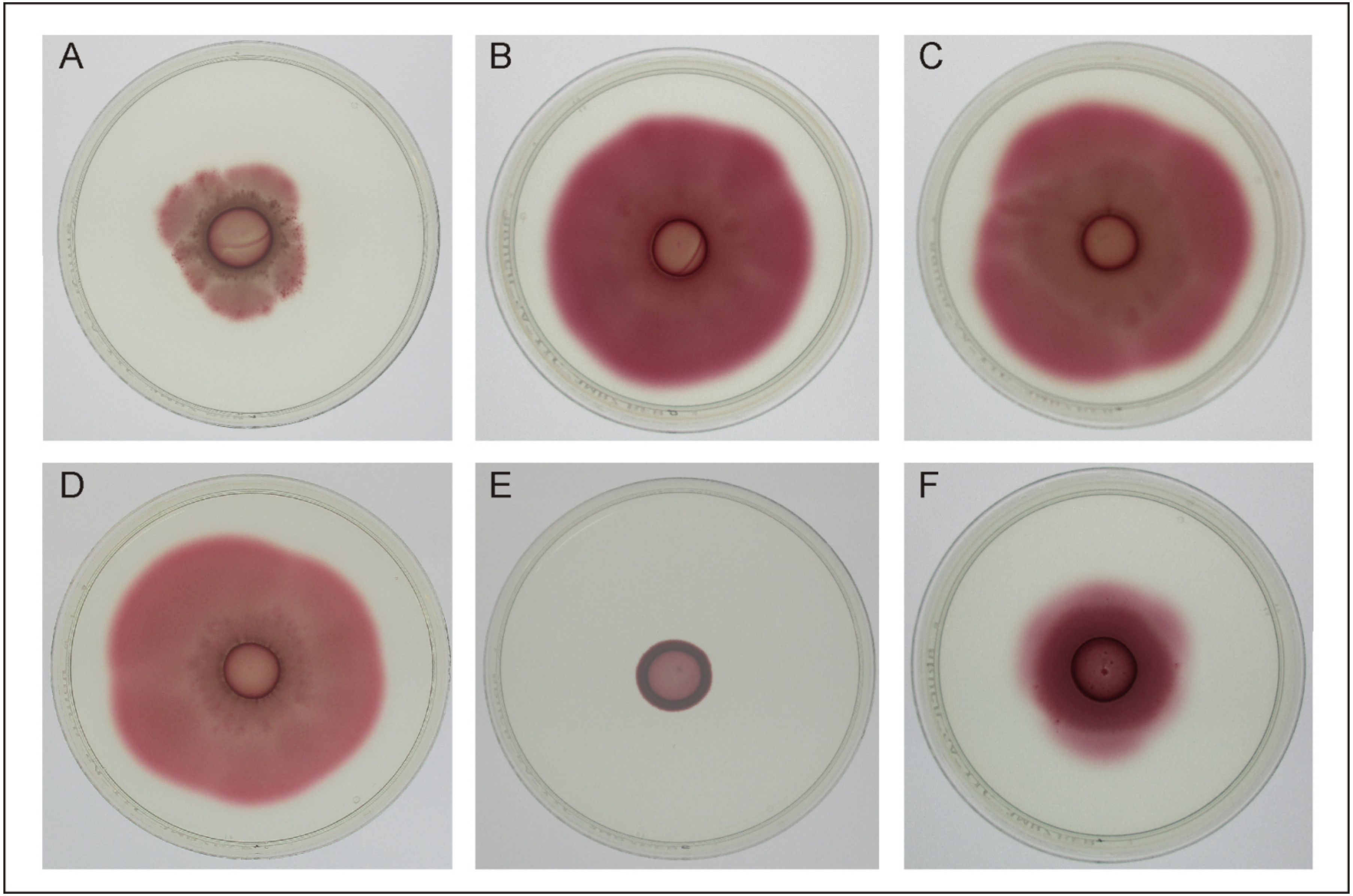

Suppl. Figure S3

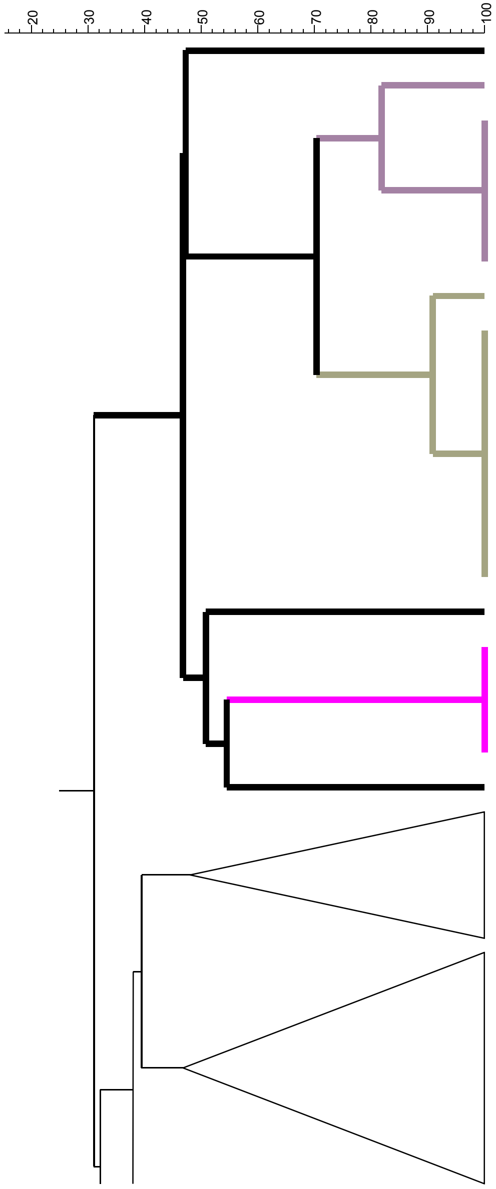

	Panel 1								Panel 2A							
	bruce06-BRU1322	bruce08-BRU1134	bruce11-BRU211	bruce12-BRU73	bruce42-BRU424	bruce43-BRU379	bruce45-BRU233	bruce55-BRU2066	bruce18-BRU339	bruce19-BRU324	bruce21-BRU329	Strain	Species	Host	MLVA-8	MLVA-11
	1	5	22	14	3	2	5	5	30	40	8	10RB9213	*Brucella* sp.	African bullfrog	101	202
	3	6		13	4	2	5	7	15	41	8	10RB9214	*Brucella* sp.	African bullfrog	103	198
	3	6	26	13	4	2	5	7	10	41	8	10RB9212	*Brucella* sp.	African bullfrog	103	197
	3	6	26	13	4	2	5	7	10	41	8	10RB9211	*Brucella* sp.	African bullfrog	103	197
	3	6	26	13	4	2	5	7	10	41	8	09RB8910	*Brucella* sp.	African bullfrog	103	197
	3	6	26	13	4	2	5	7	10	41	8	09RB8909	*Brucella* sp.	African bullfrog	103	197
	3	6	26	13	4	2	5	7	10	41	8	09RB8908	*Brucella* sp..	African bullfrog	103	197
	1	6	23	13	3	2	5	7	9	40	8	10RB9210	*Brucella* sp.	African bullfrog	104	200
	1	6	23	13	3	2	5	7	10	40	8	10RB9217	*Brucella* sp.	African bullfrog	104	199
	1	6	23	13	3	2	5	7	10	40	8	10RB9216	*Brucella* sp.	African bullfrog	104	199
	1	6	23	13	3	2	5	7	10	40	8	10RB9209	*Brucella* sp.	African bullfrog	104	199
	1	6	23	13	3	2	5	7	10	40	8	10RB9208	*Brucella* sp.	African bullfrog	104	199
	1	6	23	13	3	2	5	7	10	40	8	10RB9207	*Brucella* sp.	African bullfrog	104	199
	1	6	23	13	3	2	5	7	10	40	8	10RB9206	*Brucella* sp.	African bullfrog	104	199
	1	6	23	13	3	2	5	7	10	40	8	10RB9205	*Brucella* sp.	African bullfrog	104	199
	1	6	23	13	3	2	5	7	10	40	8	09RB8471	*Brucella* sp.	African bullfrog	104	199
	2	4		12	4	2	5	3	10	40	9	10RB9215	*Brucella* sp.	African bullfrog	105	196
	2	5		13	4	2	5	6	9	41	8	09RB8918	*Brucella* sp.	African bullfrog	102	201
	2	5		13	4	2	5	6	9	41	8	09RB8915	*Brucella* sp.	African bullfrog	102	201
	2	5		13	4	2	5	6	9	41	8	09RB8914	*Brucella* sp.	African bullfrog	102	201
	2	5		13	4	2	5	6	9	41	8	09RB8913	*Brucella* sp.	African bullfrog	102	201
	2	5	9	13	3	2	5	4		40		BO1	*B. inopinata*	Human	82	191

B. microti

B. ceti and *B. pinnipedialis*

Suppl. Figure S4

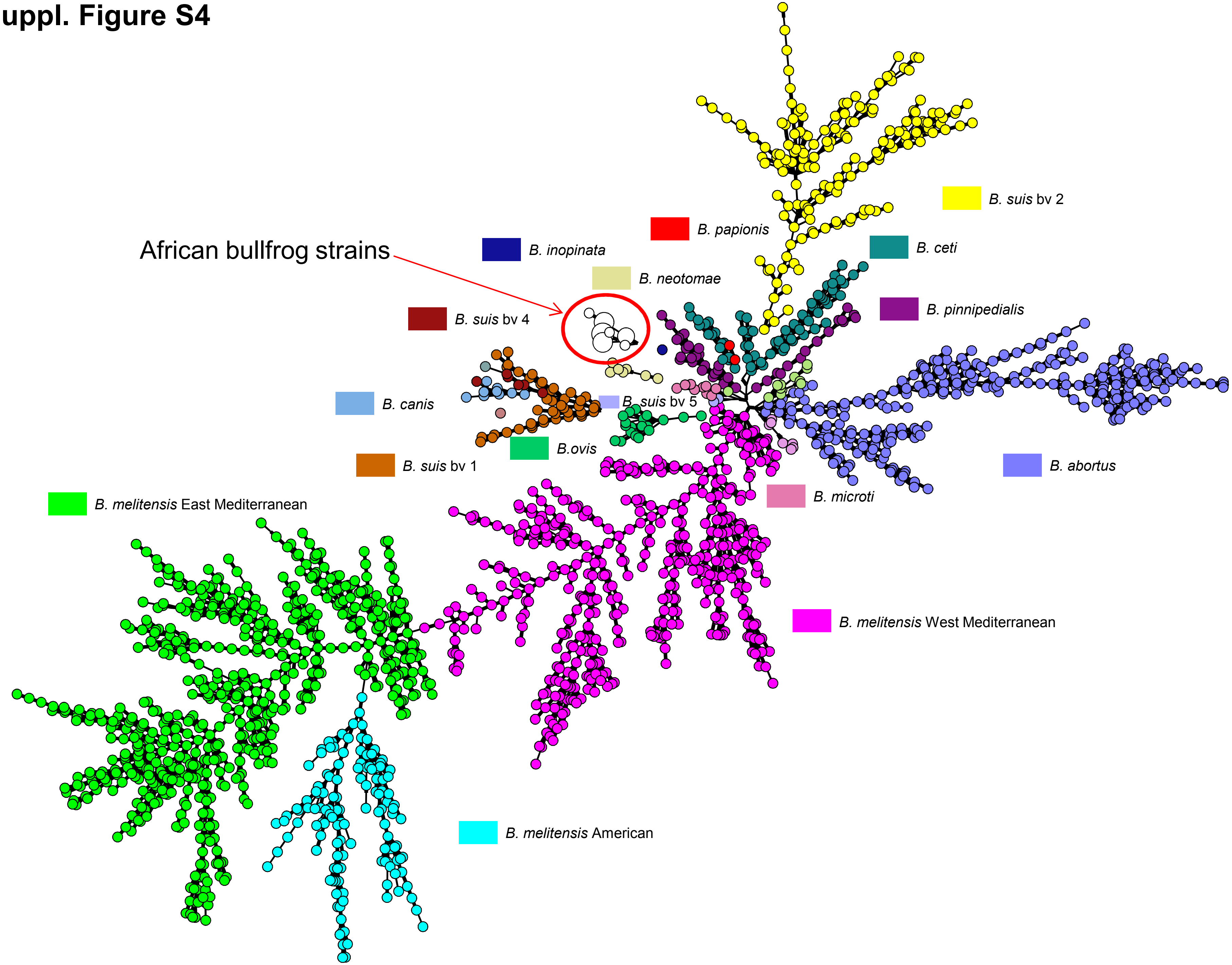

Suppl. Table S1

Substrate designation		Substrate class	*Brucella* species												African bullfrog strains					
			B. abortus	*B. melitensis*	*B. suis*	*B. ovis*	*B. canis*	*B. neotomae*	*B. ceti*	*B. pinnipedialis*	*B. microti*	*B. inopinata*	*B. papionis*	*B. vulpis*	09RB8471	09RB8910	09RB8913	09RB8918	10RB9213	10RB9215
d-Ala-pNA	DANA	aminopeptidases with pNA	+	+	+	+	+	-	+	+	+	+	-	-	+	+	+	+	+	+
Gly-pNA	GNA	aminopeptidases with pNA	+	+	+	+	+	-	+	+	+	+	-	-	+	+	+	+	+	+
Leu-pNA	LNA	aminopeptidases with pNA	-	v	v	-	-	-	v	-	+	+	+	+	-	-	+	+	+	-
Lys-pNA	KNA	aminopeptidases with pNA	v	+	v	-	v	-	+	-	+	+	+	+	+	+	+	+	+	+
Gly-Pro-pNA	GPNA	aminopeptidases with pNA	v	+	+	-	+	+	+	-	+	+	-	-	+	+	+	+	+	+
Ala-Phe-Pro-pNA	AFPNA	aminopeptidases with pNA	-	+	+	-	-	-	-	-	-	+	-	-	-	-	-	-	-	-
**Glu(pNA)-OH**	**ENAOH**	**aminopeptidases with pNA**	**-**	**-**	**-**	**-**	**-**	**-**	**-**	**-**	**-**	**-**	**-**	**-**	**-**	**-**	**-**	**-**	**-**	**-**
**Pyr-pNA**	**PYRNA**	**aminopeptidases with pNA**	**-**	**-**	**-**	**-**	**-**	**-**	**-**	**-**	**-**	**-**	**-**	**-**	**-**	**-**	**-**	**-**	**-**	**-**
**H-hydroxyproline-ßNA**	**HP**	**aminopeptidases with βNA**	**+**	**+**	**+**	**+**	**+**	**+**	**+**	**+**	**+**	**+**	**-**	**+**	**+**	**+**	**+**	**+**	**+**	**+**
Leu-ßNA	IL	aminopeptidases with βNA	-	-	-	-	-	-	-	-	-	-	-	-	-	-	-	-	-	-
Lys-ßNA	K	aminopeptidases with βNA	+	+	+	+	+	-	+	+	+	+	+	+	+	+	+	+	+	+
Arg-Arg-ßNA	RR	aminopeptidases with βNA	v	-	v	+	+	-	+	-	v	+	+	-	+	+	+	+	+	+
Gly-Gly-ßNA	GG	aminopeptidases with βNA	+	+	+	+	+	-	+	+	+	+	+	+	+	+	+	+	+	+

Pro-ßNA	P	aminopeptidases with βNA	+	+	+	+	+	+	+	+	+	+	-	+	+	+	+	+	+	+
Ac-Gly-Lys-βNA	AcGK	aminopeptidases with βNA	-	-	-	+	+	-	v	-	-	+	+	-	-	-	-	-	-	-
Ala-Phe-Pro-Ala-ßNA	AFPA	aminopeptidases with βNA	v	+	+	-	+	+	+	v	-	+	v	+	-	-	-	+	+	-
Asn-ßNA	N	aminopeptidases with βNA	v	v	+	+	+	-	-	v	-	+	-	+	+	+	+	+	+	+
Trp-βNA	W	aminopeptidases with βNA	+	+	v	-	-	+	+	+	+	+	-	-	+	+	+	+	+	+
Glu-His-ßNA	EH	aminopeptidases with βNA	+	+	+	-	-	+	-	-	+	+	-	+	+	+	+	+	+	+
Ac-Lys-Ala-ßNA	AcKA	aminopeptidases with βNA	-	v	+	+	+	+	+	-	-	+	+	-	-	-	-	-	-	-
D-Ala-D-Ala-ßNA	dAdA	aminopeptidases with βNA	+	-	v	-	-	-	+	v	+	v	-	-	+	+	+	+	+	+
Val-ßNA	V	aminopeptidases with βNA	-	-	v	-	-	-	-	-	-	-	-	-	-	-	-	+	+	+
Ile-ßNA	I	aminopeptidases with βNA	-	v	v	-	-	-	-	v	-	-	-	-	-	-	-	-	+	-
Val-Tyr-Ser-ßNA	VTS	aminopeptidases with βNA	-	v	+	+	-	-	-	-	-	+	-	+	-	-	-	-	+	-
Asp-ßNA	D	aminopeptidases with βNA	-	-	-	-	-	-	-	-	-	-	-	-	-	-	-	-	-	-
Glu-ßNA	E	aminopeptidases with βNA	-	-	-	-	-	-	-	-	-	-	-	-	-	-	-	-	-	-
Pyr-ßNA	Pyr	aminopeptidases with βNA	-	-	-	-	-	-	-	-	-	-	-	-	-	-	-	-	-	-
ß-Ala-ßNA	βA	aminopeptidases with βNA	-	-	-	-	-	-	-	-	-	-	-	-	-	-	-	-	-	-
H-Val-4-MßNA	V4M	aminopeptidases with βNA	-	-	-	-	-	-	-	-	-	-	-	-	-	-	-	-	-	-
bis-p-nitrophenyl phosphate	BISPH7	phosphatases	-	-	v	-	-	-	-	-	+	+	-	-	+	+	+	+	+	+
p-nitrophenyl phosphate di(2-amino-2-ethyl-1,3-propanediol)	PHOS7	phosphatases	-	-	-	-	-	-	-	-	+	+	-	-	+	+	+	+	+	+
p-nitrophenyl-a-d-glucopyranoside	aGLU7	glucosidases	-	-	+	-	v	+	-	-	+	+	-	-	+	+	+	+	+	+
p-nitrophenyl-a-d-maltoside	aMAL7	glucosidases	-	-	-	-	-	-	-	-	+	+	-	-	+	+	+	+	+	+

p-nitrophenyl-a-d-glucopyranoside	aGLU5	glucosidases	-	-	v	-	-	-	-	-	+	+	-	-	+	+	+	+	+	+
p-nitrophenyl-a-d-xylopyranoside	aXYL7	glucosidases	-	-	+	-	-	-	-	-	+	+	-	-	+	+	+	+	+	+
p-nitrophenyl-n-acetyl-ß-D-glucosaminide	CHIT7	esterases	-	-	v	-	-	-	-	-	+	+	-	+	+	+	+	+	+	+
i-erythritol	EROL	sugar derivates	+	+	v	-	-	+	v	-	+	v	-	v	-	-	-	-	+	-
L(-)-fucose	L-FUC	monosaccharides	-	v	-	-	v	-	v	-	+	+	-	-	+	+	-	-	+	-
D-glucose-L-cysteine	GLUCY	sugar derivates	v	v	+	-	-	+	v	v	+	+	v	-	+	+	-	-	+	-
D(-)-ribose	D-RIB	monosaccharides	v	-	v	-	+	-	-	-	+	v	-	-	-	-	-	-	+	-
D(-)-arabinose	D-ARA	monosaccharides	-	v	-	-	-	-	-	-	v	v	-	-	-	+	-	-	-	-
D(+)-glucose	D-GLU	monosaccharides	-	v	v	-	-	+	-	-	+	v	-	-	-	+	-	-	+	-
L(+)-arabinose	L-ARA	monosaccharides	v	-	v	-	-	+	v	-	+	+	-	-	+	+	-	-	+	+
2-deoxy-D-galactose	DOGAL	monosaccharides	-	-	-	-	-	-	-	-	-	-	-	v	-	-	-	-	-	-
D(+)-galactose	D-GAL	monosaccharides	v	-	v	-	-	-	-	-	+	+	-	-	-	+	-	-	+	-
D(+)-xylose	D-XYL	monosaccharides	-	-	v	-	-	-	-	-	+	-	-	-	-	-	-	-	-	-
a-D-talose	D-TAL	monosaccharides	+	+	+	-	-	+	+	-	+	-	-	-	-	+	-	-	+	+
D-threitol	D-TOL	sugar derivates	+	-	v	v	+	-	+	-	+	+	-	-	-	+	-	-	+	+
adonite	ADON	monosaccharides	+	+	v	v	-	+	+	+	+	-	+	-	-	-	-	-	+	+
sucrose	SUCR	disaccharides	-	-	+	-	+	-	-	-	+	+	-	-	+	+	-	-	+	+
iso-maltose	MALTO	disaccharides	-	-	+	-	+	-	-	v	+	+	-	-	+	+	+	+	+	+
D(-)-fructose	D-FRU	monosaccharides	v	+	+	-	+	+	+	-	+	+	+	+	+	+	+	-	+	+
1-deoxy-1-nitro-D-sorbitol	DNSOL	sugar derivates	-	-	-	-	-	-	-	-	-	-	-	-	-	-	-	-	-	-
D(-)-lyxosylamine	LYXA	sugar derivates	-	-	+	-	+	+	+	-	+	+	+	v	+	+	+	+	+	+

palatinose	PAL	disaccharides	-	-	+	-	+	-	-	-	+	+	-	-	+	+	-	-	+	+
myo-inositol	INOL	sugar derivates	-	-	-	-	-	-	-	-	-	+	-	v	-	+	+	-	+	+
inosine	INON	sugar derivates	-	-	v	-	+	-	+	-	+	-	-	+	-	+	+	-	+	+
gallic acid	Galli	organic acids	-	+	+	-	+	+	v	-	+	v	+	-	-	-	-	-	-	-
DL-lactic acid	dlLac	organic acids	-	-	+	-	-	-	-	-	+	v	-	-	-	-	-	-	+	-
acetate	Acet	amino acid derivates	-	-	+	+	+	+	-	-	+	+	+	v	+	+	+	+	+	+
L-asparagine	L-Asn	amino acids	+	+	v	-	-	-	-	-	+	+	-	v	+	+	+	+	+	+
L-glutamic acid	L-Glu	amino acids	v	+	v	-	v	+	-	-	+	+	-	-	+	+	+	-	+	+
ala-gln	AlaGln	amino acids	v	-	-	-	-	-	-	-	+	v	-	-	-	+	-	-	+	-
guanidinosuccinic acid	GuaSu	organic acids	v	-	v	-	v	-	-	-	+	v	-	-	+	+	-	-	+	+
L-cystine	L-Cyss	amino acids	+	+	v	+	-	-	v	+	+	-	v	v	+	+	+	-	-	-
D-alanine	D-Ala	amino acids	v	+	v	-	-	+	-	-	+	+	-	-	+	+	+	-	+	+
propionic acid	Propn	organic acids	-	+	v	-	-	+	v	-	-	+	-	-	-	-	-	-	+	-
L-alanine	L-Ala	amino acids	+	+	v	-	v	-	-	-	+	+	-	-	+	+	+	-	+	+
DL-ß-hydroxybutyric acid	ßHBut	organic acids	-	-	+	-	+	+	+	-	+	+	+	-	-	-	-	-	-	-
D-asparagine	D-Asn	amino acids	v	+	-	-	-	+	-	-	-	v	-	-	+	+	+	-	+	-
L-arginine	L-Arg	amino acids	-	-	+	-	v	-	-	-	+	+	-	v	+	+	+	-	+	+
Na-acetyl-l-arginine	AcArg	amino acid derivates	-	-	+	-	+	-	-	-	v	+	-	v	+	+	+	+	+	+
glyoxylic acid	Glyx	organic acids	-	-	-	-	-	-	-	-	-	+	-	v	-	-	-	-	-	-
L-serine	L-Ser	amino acids	-	v	-	-	-	-	-	-	+	+	-	-	+	+	+	-	+	+
hippuryl-arg	HipArg	peptides	-	-	v	-	v	-	-	-	v	+	-	-	+	+	+	+	+	+
L-carnosine	L-Caro	amino acids	-	-	v	-	-	-	-	-	+	+	-	-	+	+	+	-	+	+

glycine	Gly	amino acids	-	-	-	-	-	-	-	-	+	+	-	-	+	+	+	-	+	-
adipic acid	Adipa	organic acids	-	-	v	-	+	-	+	-	+	+	-	-	+	+	+	+	+	+
L-proline	L-Pro	amino acids	-	+	-	-	-	-	-	-	+	+	-	-	+	+	+	+	+	+
D-proline	D-Pro	amino acids	-	+	-	-	-	-	-	-	+	+	-	-	+	+	+	-	+	+
fumaric acid	Fuma	organic acids	-	-	-	-	-	-	-	-	+	+	-	-	+	+	+	+	+	+
D-serine	D-Ser	amino acids	-	+	-	-	-	-	-	-	-	-	-	v	-	-	-	-	-	-
glycolic acid	Glyc	organic acids	-	-	-	-	-	-	-	-	-	+	-	-	+	+	+	-	+	+
Adenine	Adeni	amino acid derivates	+	+	v	-	+	-	+	v	-	-	+	v	-	+	+	-	-	-
glutaric acid	Gluta	organic acids	-	-	-	-	-	-	-	-	+	+	v	-	+	+	-	-	+	+
mesaconic acid	Mesac	organic acids	-	-	-	-	-	-	-	-	+	-	-	-	-	-	-	-	-	-
D-histidine	D-His	amino acids	-	-	-	-	-	-	-	-	-	+	-	-	+	+	+	-	+	+
nitrite	NTI	classical reactions	-	-	-	-	-	-	-	-	+	+	-	-	+	+	+	+	+	+
nitrate	NTA	classical reactions	+	v	+	-	v	-	+	+	+	v	-	-	-	-	-	-	-	-
pyrazinamidase	PCA	classical reactions	v	+	v	-	-	+	+	+	+	v	+	v	+	+	+	+	-	+
Voges Proskauer	VP	classical reactions	+	+	v	-	+	+	+	+	+	+	+	+	-	+	+	+	+	+
**urease**	**urease**	**classical reactions**	**+**	**+**	**+**	**+**	**+**	**+**	**+**	**+**	**+**	**+**	**+**	**+**	**+**	**+**	**+**	**+**	**+**	**+**
hydrogen sulfide	H2S	classical reactions	-	-	v	-	-	-	-	-	+	+	-	-	+	+	+	+	+	+

Suppl. Table S2

Strain	Accession	PATRIC ID	Start	End	Length	AA Length	Strand	PATRIC genus-specific families (PLfams)	Product	09RB8471	09RB8910	09RB8913	10RB9213	10RB9215
	234.101.con.0001	peg.208	199942	200112	171	56	-	PLF_234_00007048	hypothetical protein					
	234.101.con.0001	peg.272	257293	257556	264	87	+	PLF_234_00006633	hypothetical protein					
	234.101.con.0001	peg.741	706589	706897	309	102	+	PLF_234_00006818	FIG032766: hypothetical protein					
	234.101.con.0001	peg.882	859448	859624	177	58	-	PLF_234_00003752	hypothetical protein					
	234.101.con.0001	peg.917	895161	895301	141	46	-	PLF_234_00005331	hypothetical protein					
	234.101.con.0001	peg.1574	1533698	1534393	696	231	+	PLF_234_00007031	hypothetical protein					
	234.101.con.0001	peg.1659	1612515	1612745	231	76	-	PLF_234_00006933	membrane protein, putative					
09RB8471	234.101.con.0001	peg.1865	1814210	1814626	417	138	+	PLF_234_00006794	hypothetical protein					
	234.101.con.0001	peg.1866	1814623	1815009	387	128	+	PLF_234_00006659	hypothetical protein					
	234.101.con.0001	peg.1901	1841903	1842064	162	53	+	PLF_234_00006958	hypothetical protein					
	234.101.con.0001	peg.1979	1918235	1918444	210	69	-	PLF_234_00006630	hypothetical protein					
	234.101.con.0001	peg.2077	2003776	2003892	117	38	+	PLF_234_00006505	hypothetical protein					
	234.101.con.0002	peg.2954	683289	683612	324	107	-	PLF_234_00007136	hypothetical protein					
	234.101.con.0002	peg.3127	865844	866005	162	53	-	PLF_234_00007102	hypothetical protein					
	234.101.con.0002	peg.3277	1018786	1019508	723	240	+	PLF_234_00007031	hypothetical protein					
	234.102.con.0001	peg.175	162802	162948	147	48	+	PLF_234_00007077	hypothetical protein					
	234.102.con.0001	peg.644	610668	610787	120	39	-	PLF_234_00006890	hypothetical protein					
	234.102.con.0001	peg.673	636805	636975	171	56	-	PLF_234_00005526	FIG00451131: hypothetical protein					
	234.102.con.0001	peg.765	731061	731912	852	283	+	PLF_234_00007149	Pyrimidine ABC transporter, ATP-binding protein					
	234.102.con.0001	peg.766	731946	732839	894	297	+	PLF_234_00007059	Pyrimidine ABC transporter, transmembrane component 1					
	234.102.con.0001	peg.767	732836	733702	867	288	+	PLF_234_00006989	Pyrimidine ABC transporter, transmembrane component 2					
	234.102.con.0001	peg.768	733801	734799	999	332	+	PLF_234_00006746	Pyrimidine ABC transporter, substrate-binding component					
	234.102.con.0001	peg.790	755338	755646	309	102	+	PLF_234_00006818	FIG032766: hypothetical protein					
	234.102.con.0001	peg.935	907044	907220	177	58	-	PLF_234_00003752	hypothetical protein					
09RB8910	234.102.con.0001	peg.953	920442	920597	156	51	-	PLF_234_00003113	hypothetical protein					
	234.102.con.0001	peg.1523	1489232	1489399	168	55	-	PLF_234_00003970	hypothetical protein					
	234.102.con.0001	peg.1797	1727373	1727549	177	58	-	PLF_234_00003604	FIG00450737: hypothetical protein					
	234.102.con.0001	peg.1851	1777595	1777750	156	51	-	PLF_234_00006879	hypothetical protein					
	234.102.con.0001	peg.1880	1807044	1807556	513	170	-	PLF_234_00006641	hypothetical protein					
	234.102.con.0001	peg.1990	1918434	1918562	129	42	+	PLF_234_00005320	hypothetical protein					
	234.102.con.0001	peg.2076	2001100	2001309	210	69	-	PLF_234_00006630	hypothetical protein					
	234.102.con.0001	peg.2254	2167892	2168026	135	44	+	PLF_234_00007061	hypothetical protein					
	234.102.con.0002	peg.2444	75747	76325	579	192	+	PLF_234_00006687	hypothetical protein					
	234.102.con.0002	peg.2445	76570	76758	189	62	+	PLF_234_00006973	hypothetical protein					
	234.102.con.0002	peg.2638	286377	286511	135	44	-	PLF_234_00006884	hypothetical protein					
	234.102.con.0002	peg.2752	425261	425662	402	133	+	PLF_234_00006702	FIG00450865: hypothetical protein					
	234.102.con.0002	peg.3174	860241	860450	210	69	+	PLF_234_00003836	hypothetical protein					
	234.106.con.0001	peg.2	459	1181	723	240	+	PLF_234_00007031	hypothetical protein					
	234.106.con.0003	peg.88	26645	26848	204	67	+	PLF_234_00005342	FIG00450274: hypothetical protein					
	234.106.con.0005	peg.449	6795	6956	162	53	-	PLF_234_00007102	hypothetical protein					
	234.106.con.0009	peg.833	59473	59643	171	56	-	PLF_234_00005526	FIG00451131: hypothetical protein					
	234.106.con.0009	peg.921	152751	153602	852	283	+	PLF_234_00007149	Pyrimidine ABC transporter, ATP-binding protein					
	234.106.con.0009	peg.922	153636	154529	894	297	+	PLF_234_00007059	Pyrimidine ABC transporter, transmembrane component [illegible]					
	234.106.con.0009	peg.923	154526	155392	867	288	+	PLF_234_00006989	Pyrimidine ABC transporter, transmembrane component [illegible]					
	234.106.con.0009	peg.924	155491	156489	999	332	+	PLF_234_00006746	Pyrimidine ABC transporter, substrate-binding component					
	234.106.con.0010	peg.1017	89738	89863	126	41	+	PLF_234_00006651	hypothetical protein					
	234.106.con.0010	peg.1101	171127	171435	309	102	-	PLF_234_00006818	FIG032766: hypothetical protein					
	234.106.con.0017	peg.1420	71129	71788	660	219	-	PLF_234_00007045	Type II restriction enzyme NspV (EC 3.1.21.4) (Endonuclease NspV) (R.NspV)					
	234.106.con.0017	peg.1506	161674	161892	219	72	-	PLF_234_00004996	hypothetical protein					
	234.106.con.0021	peg.1585	3008	3703	696	231	-	PLF_234_00004367	hypothetical protein					
	234.106.con.0021	peg.1586	3691	4188	498	165	-	PLF_234_00004869	hypothetical protein					
	234.106.con.0021	peg.1587	4185	4832	648	215	-	PLF_234_00004868	Phage protein					
	234.106.con.0021	peg.1590	5358	5753	396	131	-	PLF_234_00004656	hypothetical protein					
	234.106.con.0021	peg.1593	6453	6674	222	73	-	PLF_234_00004864	hypothetical protein					
	234.106.con.0021	peg.1595	7038	7481	444	147	-	PLF_234_00004862	hypothetical protein					
	234.106.con.0021	peg.1597	8103	8816	714	237	-	PLF_234_00004861	hypothetical protein					
	234.106.con.0021	peg.1600	9415	10206	792	263	-	PLF_234_00004860	DNA adenine methyltransferase					
	234.106.con.0021	peg.1602	11345	11719	375	124	-	PLF_234_00005722	hypothetical protein					
	234.106.con.0021	peg.1603	11716	12372	657	218	-	PLF_234_00004859	hypothetical protein					
	234.106.con.0021	peg.1606	13758	13961	204	67	+	PLF_234_00005007	hypothetical protein					
	234.106.con.0021	peg.1610	14934	15131	198	65	+	PLF_234_00004857	hypothetical protein					
	234.106.con.0021	peg.1612	15361	15966	606	201	+	PLF_234_00004856	Terminase small subunit					
	234.106.con.0021	peg.1613	15969	17495	1527	508	+	PLF_234_00004657	putative terminase large subunit protein					
09RB8913	234.106.con.0021	peg.1614	17576	18007	432	143	+	PLF_234_00004855	hypothetical protein					
	234.106.con.0021	peg.1615	18000	20318	2319	772	+	PLF_234_00004854	Phage portal protein					
	234.106.con.0021	peg.1616	20368	21363	996	331	+	PLF_234_00004655	hypothetical protein					
	234.106.con.0021	peg.1620	23248	24351	1104	367	+	PLF_234_00004853	Phage protein					
	234.106.con.0021	peg.1621	24415	24843	429	142	+	PLF_234_00004852	hypothetical protein					
	234.106.con.0021	peg.1622	24943	25401	459	152	+	PLF_234_00004654	hypothetical protein					
	234.106.con.0021	peg.1623	25415	26101	687	228	+	PLF_234_00004851	hypothetical protein					
	234.106.con.0021	peg.1624	26101	26733	633	210	+	PLF_234_00004850	Phage protein					
	234.106.con.0021	peg.1625	26741	27157	417	138	+	PLF_234_00004849	hypothetical protein					
	234.106.con.0021	peg.1627	27996	28277	282	93	+	PLF_234_00004848	hypothetical protein					
	234.106.con.0021	peg.1634	33763	34023	261	86	+	PLF_234_00004847	hypothetical protein					
	234.106.con.0021	peg.1635	34020	34433	414	137	+	PLF_234_00004846	hypothetical protein					
	234.106.con.0021	peg.1636	34430	34867	438	145	+	PLF_234_00004937	hypothetical protein					
	234.106.con.0021	peg.1638	35255	36427	1173	390	+	PLF_234_00004354	Phage protein					
	234.106.con.0021	peg.1639	36424	37608	1185	394	+	PLF_234_00004845	hypothetical protein					

	234.106.con.0021	peg.1641	38135	53020	14886	4961	+	PLF_234_00004568	Cell wall hydrolyses involved in spore germination
	234.106.con.0021	peg.1643	53243	53902	660	219	+	PLF_234_00004583	Lysozyme (N-acetylmuramidase) family, (EC 3.2.1.17)
	234.106.con.0021	peg.1644	53899	54420	522	173	+	PLF_234_00004844	hypothetical protein
	234.106.con.0021	peg.1647	55150	55896	747	248	-	PLF_234_00005838	hypothetical protein
	234.106.con.0021	peg.1648	56412	57479	1068	355	-	PLF_234_00004598	Plasmid replication protein RepB
	234.106.con.0026	peg.2160	2771	3115	345	114	-	PLF_234_00007136	hypothetical protein
	234.106.con.0029	peg.2408	27787	27915	129	42	+	PLF_234_00004834	hypothetical protein
	234.106.con.0036	peg.2691	13739	13906	168	55	-	PLF_234_00003970	hypothetical protein
	234.106.con.0037	peg.2749	95	277	183	60	-	PLF_234_00006892	hypothetical protein
	234.106.con.0041	peg.2893	21048	21176	129	42	+	PLF_234_00006992	hypothetical protein
	234.106.con.0041	peg.2898	24544	24753	210	69	-	PLF_234_00006630	hypothetical protein
	234.106.con.0072	peg.3245	2	514	513	171	-	PLF_234_00004842	Phage DNA replication protein
	234.106.con.0072	peg.3246	578	1564	987	328	-	PLF_234_00004913	Phage minor capsid protein - DNA pilot protein
	234.106.con.0072	peg.3248	2211	3494	1284	427	-	PLF_234_00005551	Phage major capsid protein
	234.106.con.0072	peg.3250	4359	5384	1026	341	-	PLF_234_00004842	Phage DNA replication protein
	234.106.con.0080	peg.3278	429	578	150	49	-	PLF_234_00006876	hypothetical protein
**10RB9213**	234.105.con.0002	peg.340	328251	328385	135	44	+	PLF_234_00006798	hypothetical protein
	234.105.con.0003	peg.574	195568	195876	309	102	-	PLF_234_00006818	FIG032766: hypothetical protein
	234.105.con.0003	peg.613	226454	226561	108	35	+	PLF_234_00006703	hypothetical protein
	234.105.con.0003	peg.706	327467	327637	171	56	+	PLF_234_00005526	FIG00451131: hypothetical protein
	234.105.con.0004	peg.808	76271	76447	177	58	+	PLF_234_00003604	FIG00450737: hypothetical protein
	234.105.con.0005	peg.1175	337393	337602	210	69	-	PLF_234_00003836	hypothetical protein
	234.105.con.0006	peg.1468	177242	177364	123	40	+	PLF_234_00003423	hypothetical protein
	234.105.con.0006	peg.1472	179719	179928	210	69	-	PLF_234_00006630	hypothetical protein
	234.105.con.0007	peg.1550	82506	82829	324	107	+	PLF_234_00006859	FIG00450089: hypothetical protein
	234.105.con.0009	peg.1751	131739	131855	117	38	-	PLF_234_00006895	hypothetical protein
	234.105.con.0010	peg.1908	48800	49015	216	71	+	PLF_234_00006780	hypothetical protein
	234.105.con.0014	peg.2399	154248	154412	165	54	-	PLF_234_00006898	hypothetical protein
	234.105.con.0020	peg.2891	62698	62829	132	43	-	PLF_234_00004880	hypothetical protein
	234.105.con.0031	peg.3352	2826	3893	1068	355	+	PLF_234_00004598	Plasmid replication protein RepB
	234.105.con.0031	peg.3353	4409	5155	747	248	+	PLF_234_00005838	hypothetical protein
	234.105.con.0031	peg.3356	5885	6406	522	173	-	PLF_234_00004844	hypothetical protein
	234.105.con.0031	peg.3357	6403	7062	660	219	-	PLF_234_00004583	Lysozyme (N-acetylmuramidase) family, (EC 3.2.1.17)
	234.105.con.0031	peg.3358	7285	22170	14886	4961	-	PLF_234_00004568	Cell wall hydrolyses involved in spore germination
	234.105.con.0031	peg.3360	22697	23881	1185	394	-	PLF_234_00004845	hypothetical protein
	234.105.con.0031	peg.3361	23878	25050	1173	390	-	PLF_234_00004354	Phage protein
	234.105.con.0031	peg.3363	25438	25875	438	145	-	PLF_234_00004937	hypothetical protein
	234.105.con.0031	peg.3364	25872	26285	414	137	-	PLF_234_00004846	hypothetical protein
	234.105.con.0031	peg.3365	26282	26542	261	86	-	PLF_234_00004847	hypothetical protein
	234.105.con.0031	peg.3370	30979	31260	282	93	-	PLF_234_00004848	hypothetical protein
	234.105.con.0031	peg.3372	32099	32515	417	138	-	PLF_234_00004849	hypothetical protein
	234.105.con.0031	peg.3373	32523	33155	633	210	-	PLF_234_00004850	Phage protein
	234.105.con.0031	peg.3374	33155	33841	687	228	-	PLF_234_00004851	hypothetical protein
	234.105.con.0031	peg.3375	33855	34313	459	152	-	PLF_234_00004654	hypothetical protein
	234.105.con.0031	peg.3376	34413	34841	429	142	-	PLF_234_00004852	hypothetical protein
	234.105.con.0031	peg.3377	34905	36008	1104	367	-	PLF_234_00004853	Phage protein
	234.105.con.0031	peg.3381	37893	38888	996	331	-	PLF_234_00004655	hypothetical protein
	234.105.con.0031	peg.3382	38938	41256	2319	772	-	PLF_234_00004854	Phage portal protein
	234.105.con.0031	peg.3383	41249	41680	432	143	-	PLF_234_00004855	hypothetical protein
	234.105.con.0031	peg.3384	41761	43287	1527	508	-	PLF_234_00004657	putative terminase large subunit protein
	234.105.con.0031	peg.3385	43290	43895	606	201	-	PLF_234_00004856	Terminase small subunit
	234.105.con.0031	peg.3387	44125	44322	198	65	-	PLF_234_00004857	hypothetical protein
	234.105.con.0031	peg.3391	45295	45498	204	67	-	PLF_234_00005007	hypothetical protein
	234.105.con.0031	peg.3393	46392	46658	267	88	+	PLF_234_00004979	hypothetical protein
	234.105.con.0031	peg.3395	47335	48009	675	224	+	PLF_234_00004858	hypothetical protein
	234.105.con.0031	peg.3396	48009	48689	681	226	+	PLF_234_00004859	hypothetical protein
	234.105.con.0031	peg.3397	48686	49060	375	124	+	PLF_234_00005722	hypothetical protein
	234.105.con.0031	peg.3402	51586	52302	717	238	+	PLF_234_00004861	hypothetical protein
	234.105.con.0031	peg.3404	52924	53367	444	147	+	PLF_234_00004862	hypothetical protein
	234.105.con.0031	peg.3406	53731	53952	222	73	+	PLF_234_00004864	hypothetical protein
	234.105.con.0031	peg.3409	54652	55047	396	131	+	PLF_234_00004656	hypothetical protein
	234.105.con.0031	peg.3412	55573	56220	648	215	+	PLF_234_00004868	Phage protein
	234.105.con.0031	peg.3413	56217	56714	498	165	+	PLF_234_00004869	hypothetical protein
	234.105.con.0031	peg.3414	56702	57397	696	231	+	PLF_234_00004367	hypothetical protein
	234.105.con.0040	peg.3454	3	983	981	326	+	PLF_234_00005551	Phage major capsid protein
	234.105.con.0040	peg.3456	1630	2616	987	328	+	PLF_234_00004913	Phage minor capsid protein - DNA pilot protein
	234.105.con.0040	peg.3457	2680	4221	1542	513	+	PLF_234_00004842	Phage DNA replication protein
	234.105.con.0040	peg.3459	5086	5385	300	100	+	PLF_234_00005551	Phage major capsid protein
	234.105.con.0049	peg.3473	2	349	348	116	-	PLF_234_00007064	Mobile element protein
**10RB9215**	234.104.con.0001	peg.77	73011	73181	171	56	+	PLF_234_00005526	FIG00451131: hypothetical protein
	234.104.con.0001	peg.105	99161	99376	216	71	+	PLF_234_00006890	hypothetical protein
	234.104.con.0001	peg.439	433576	433719	144	47	+	PLF_234_00006233	hypothetical protein
	234.104.con.0001	peg.511	502038	502289	252	83	-	PLF_234_00006919	hypothetical protein
	234.104.con.0001	peg.799	793074	793202	129	42	-	PLF_234_00006972	hypothetical protein
	234.104.con.0001	peg.850	847606	847881	276	91	+	PLF_234_00006780	hypothetical protein
	234.104.con.0001	peg.938	925857	926066	210	69	+	PLF_234_00006630	hypothetical protein
	234.104.con.0001	peg.1233	1207690	1207845	156	51	+	PLF_234_00006879	hypothetical protein
	234.104.con.0001	peg.1285	1256645	1256821	177	58	+	PLF_234_00003604	FIG00450737: hypothetical protein
	234.104.con.0001	peg.1524	1493406	1493693	288	95	-	PLF_234_00004743	FIG00363499: hypothetical protein
	234.104.con.0001	peg.2205	2178237	2178545	309	102	-	PLF_234_00006818	FIG032766: hypothetical protein
	234.104.con.0002	peg.2652	381188	381511	324	107	-	PLF_234_00007136	hypothetical protein

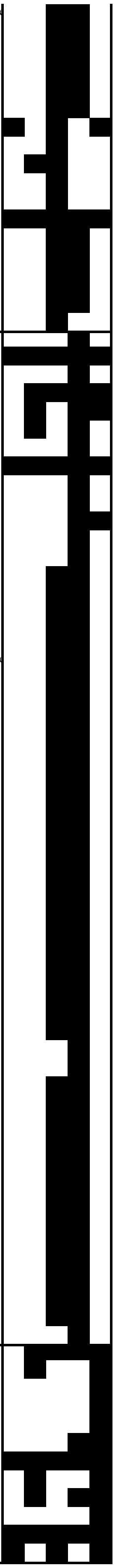

Suppl. Table S3

Identifier	PATRIC genus specific protein family identifier	Product Description	10RB9215	B13-0095	10RB9213	BO2	09RB8913	09RB8910	09RB8471	microti	BO1	
	PLF_234_00002397	Ureidoglycolate lyase (EC 4.3.2.3)	peg.208	peg.1415	peg.3137	peg.2137	peg.423	peg.538	peg.476	peg.1669	peg.1877	
	PLF_234_00001810	5-hydroxyisourate hydrolase (EC 3.5.2.17)	peg.209	peg.1416	peg.3138	peg.2136	peg.422	peg.537	peg.475	peg.1670	peg.1878	Flanking Region
	PLF_234_00001574	FIG00451437: hypothetical protein	peg.210	peg.1417	peg.3139	peg.2135	peg.421	peg.536	peg.474	peg.1671	peg.1879	
wbkD	PLF_234_00000966	Epimerase/dehydratase WbkD	peg.211	peg.1418	peg.3140	peg.2134	peg.420	peg.535	peg.473	peg.1672	peg.1880	
wbkF	PLF_234_00002829	Undecaprenyl-glycosyltransferase WbkF	peg.212	peg.1419	peg.3141	peg.2133	peg.419	peg.534	peg.472	peg.1673	peg.1881	
	PLF_234_00003531	hypothetical protein							peg.471	peg.1674	peg.1882	
		tRNA-Gln-TTG	rna.1	rna.20	rna.46	rna.38	.rna.5	pegrna.9	rna.10	rna.23	rna.33	
B13-1	PLF_234_00006756	hypothetical protein		peg.1420								
9213-1		hypothetical protein			peg.3142							
9213-2		hypothetical protein			peg.3143							
9213-3		Glycosyltransferase			peg.3144							
9213-4		hypothetical protein			peg.2425							
9213-5		hypothetical protein			peg.2426							
8910-1		hypothetical protein						peg.533				
8910-2		hypothetical protein						peg.531				
rfbD		O-antigen export system permease protein RfbD	peg.214	peg.1422	peg.2427	peg.2132		peg.530				
TagH		Teichoic acid export ATP-binding protein TagH (EC 3.6.3.40)	peg.215	peg.1423	peg.2428	peg.2131		peg.529				
		Glycosyltransferase			peg.2429							
		UDP-N-acetylglucosamine 2-epimerase (EC 5.1.3.14)			peg.2430							
		Possible glycosyltransferase			peg.2431							
	PLF_234_00005214	hypothetical protein	peg.216	peg.1424								
		Glycosyl transferase, family 2	peg.219	peg.1426	peg.2432							
BO2-1	PLF_234_00005216	hypothetical protein				peg.2130						
BO-2	PLF_234_00005215	Glycosyl transferase, group 2 family				peg.2129						
BO2-3	PLF_234_00005589	putative acetyl transferase by domain				peg.2128						
BO2-4		hypothetical protein				peg.2127						
8913-1		FIG01202174: hypothetical protein					peg.418					
8913-2		FIG01201580: hypothetical protein					peg.417					
8913-3		hypothetical protein					peg.416					
8913-4		O-antigen export system, ATP-binding protein					peg.415					
8913-5		FIG01016064: hypothetical protein					peg.414					
8913-6		hypothetical protein					peg.413					
8913-7		hypothetical protein					peg.412					
8913-8		Probable dTDP-4-dehydrorhamnose reductase (EC 1.1.1.133)					peg.411					
8913-9		Capsular polysaccharide biosynthesis protein CapD					peg.410					
8913-10		UDP-N-acetyl-L-fucosamine synthase (EC 5.1.3.28)					peg.409					
8910-3		hypothetical protein						peg.528				
8910-4		hypothetical protein						peg.527				
8910-5		hypothetical protein						peg.526				
8910-6		hypothetical protein						peg.525				
8910-7		hypothetical protein						peg.524				All WBK genes
8910-8		hypothetical protein						peg.519				
8910-9		Glycosyl transferase, family 2						peg.518				
8910-10		hypothetical protein						peg.517				
8471-1		hypothetical protein							peg.470			

8471-2		Aldehyde dehydrogenase B (EC 1.2.1.22)							peg.469			
8471-3		Lipid carrier : UDP-N-acetylgalactosaminyltransferase (EC 2.4.1.-) / Alpha-1,3-N-acetylgalactosamine transferase PglA (EC 2.4.1.-); Putative glycosyltransferase							peg.467			
8471-4		hypothetical protein							peg.466			
8471-5		hypothetical protein							peg.465			
8471-6		Teichoic acid export ATP-binding protein TagH (EC 3.6.3.40)							peg.464			
8471-7		hypothetical protein							peg.463			
8471-8		hypothetical protein							peg.462			
8471-9		hypothetical protein							peg.461			
8471-10		hypothetical protein							peg.460			
8471-11		hypothetical protein							peg.459			
8471-12		hypothetical protein							peg.458			
8471-13		hypothetical protein							peg.457			
BO2-5		hypothetical protein				peg.2126						
rmlA	PLF_234_00005213	Glucose-1-phosphate thymidylyltransferase (EC 2.7.7.24)	peg.220	peg.2273	peg.2433	peg.2115						
rmlC	PLF_234_00005212	dTDP-4-dehydrorhamnose 3,5-epimerase (EC 5.1.3.13)	peg.221	peg.2274	peg.2434	peg.2114						
rmlB	PLF_234_00005211	dTDP-glucose 4,6-dehydratase (EC 4.2.1.46)	peg.222	peg.2275	peg.2435	peg.2113						
rmlD	PLF_234_00005210	dTDP-4-dehydrorhamnose reductase (EC 1.1.1.133)	peg.223	peg.2276	peg.2436	peg.2112						
wbkC	PLF_234_00003001	UDP-4-amino-4-deoxy-L-arabinose formyltransferase (EC 2.1.2.13) @ Formyltransferase WbkC								peg.1680	peg.1886	
wbkB	PLF_234_00003528	Perosamine synthetase WbkB								peg.1681	peg.1887	
wzt	PLF_234_00003413	O-antigen export system, ATP-binding protein								peg.1682	peg.1888	
wzm	PLF_234_00003497	O-antigen export system permease protein Wzm								peg.1683	peg.1889	
per	PLF_234_00002929	GDP-perosamine synthase (EC 2.6.1.102)								peg.1684	peg.1890	
gmd	PLF_234_00002811	GDP-mannose 4,6-dehydratase (EC 4.2.1.47)								peg.1685	peg.1891	
		hypothetical protein								peg.1686		
wbkA	PLF_234_00002490	Mannosyltransferase WbkA								peg.1692	peg.1893	
manB	PLF_234_00000736	Phosphomannomutase ManB (EC 5.4.2.8)								peg.1698	peg.1898	
manC	PLF_234_00001630	Mannose-1-phosphate guanylyltransferase (EC 2.7.7.13) / Mannose-6-phosphate isomerase (EC 5.3.1.8)								peg.1699	peg.1899	
manA	PLF_234_00000429	Mannose-6-phosphate isomerase ManA (EC 5.3.1.8)								peg.1700	peg.1900	
wbkE	PLF_234_00000443	Mannosyltransferase WbkE								peg.1701	peg.1901	
xylG	PLF_234_00000156	D-xylose transport ATP-binding protein XylG				peg.2111	peg.765	peg.516		peg.1702	peg.1902	
xylH	PLF_234_00002187	Xylose ABC transporter, permease protein XylH				peg.2110	peg.764	peg.515		peg.1703	peg.1903	
xylF	PLF_234_00000486	Xylose ABC transporter, periplasmic xylose-binding protein XylF				peg.2109	peg.763	peg.514		peg.1704	peg.1904	Flanking region
	PLF_234_00000998	Inosose isomerase (EC 5.3.99.11)	peg.224	peg.2277	peg.2437	peg.2108	peg.762	peg.513		peg.1705	peg.1906	
	PLF_234_00001018	Oxidoreductase, Gfo/Idh/MocA family	peg.225	peg.2278	peg.2438	peg.2107	peg.761	peg.512		peg.1706	peg.1907	
	PLF_234_00000688	Xylose isomerase (EC 5.3.1.5)	peg.226	peg.2279	peg.2439	peg.2106	peg.760	peg.511	peg.452	peg.1707	peg.1908	

Suppl. Table S4

	Gene	16M	*O. anthropi* AA length	*Brucella* AA Length	Amphibian isolates					Ancestral			Classic *Brucella* clade													
					9213	8913	8471	8910	9215	BO2	BO1	Aus	*microti*	*suis* bv5	*suis* bv3	*suis* bv2	*suis* bv1	*canis*	*pinnipedialis*	*ceti* B	*ceti* A	*papionis*	*ovis*	*melitensis*	*abortus*	Function
MS ring construction	FliF	BMEII0152	579	580								Ψ				Ψ				Ψ				Ψ*		MS ring and proximal rod
	FliM	BMEII1110	317	316																			Ψ		Ψ*	C ring (switch proteins)
	FliN	BMEII1112		115											Ψ											C ring (switch proteins)
	FliG	BMEII1113		351									D		Ψ	Ψ	Ψ	Ψ			Ψ	Ψ		Ψ	Ψ*	MS ring (switch)
Basal body structural proteins, facilitators and regulators	FlgB	BMEII1089		126											Ψ	Ψ										Proximal rod
	FlgC	BMEII1088		140											Ψ	Ψ										Proximal rod
	FlgF	BMEII1107		243														D						Ψ		Proximal rod
	FlaF	BMEII0162		114																						Basal body component with signal peptide for export
	FliE	BMEII1087		111																						Adaptor
	FlgG	BMEII1086		262										Ψ						Ψ		Ψ				Distal rod
	FlgJ	BMEII0170	205	196																						Cap to rod and penetration of petidoglycan layer
	FlgI	BMEII1084	413	474				409^{M}	Ψ	409^{M}	409^{M}	409^{M}	D	O	D	D	D	D	D	D	D	D	Ψ	Ψ	Ψ	P ring
	FlgH	BMEII1082		242										O	238^{M}	238^{M}	238^{M}	238^{M}		Ψ						L ring
	FlgA	BMEII1085	157	165										O								Ψ		159^{M}		periplasmic chaperone to FlgI and FlgH
	FlgE	BMEII0159	397	396																			D			Hook assembly
	FlgD	BMEII0164	136	137										289*												Hook capping protein
	FlgK	BMEII0160		484															Ψ		Ψ		D			HAP protein (at cap-replaces FlgD)
	FlgL	BMEII0161		348																						HAP protein (at cap-replaces FlgD)
	FliC	BMEII0150	327	282					307											Ψ			Ψ			Filament subunits on distal end
	FlbT	BMEII0163		152										289*												Regulates FliC
Export apparatus	FlhA	BMEII0167	696	705								Ψ				D				Ψ	D	Ψ	D	Ψ	Ψ	Core transmembrane protein-forms export channel complex inside the MS-ring structure
	FlhB	BMEII1114		356						Ψ																Core transmembrane protein-forms export channel complex inside the MS-ring structure
	FliP	BMEII1080		246										O												Core transmembrane protein-forms export channel complex inside the MS-ring structure
	FliQ	BMEII0165		88																						Core transmembrane protein-forms export channel complex inside the MS-ring structure
	FliR	BMEII0168		255												Ψ						Ψ	Ψ			Core transmembrane protein-forms export channel complex inside the MS-ring structure
	FliK	BMEII0156	416	424										Ψ						Ψ		Ψ				Substrate-specifity switching
	FliI	BMEII1105	452	453													Ψ							Ψ		Substrate-specifity switching
Motor complex	MotA	BMEII1109	290	323																						Stator protein (torque generating unit)
	MotB	BMEII0154	371	366										Ψ									Ψ			Stator protein (torque generating unit)
	FliL	BMEII1081	164	163										O		Ψ										Controls motor output and bias
	MotC	BMEII0155	431	430											Ψ	Ψ	Ψ									Acts on Mot B
	MotE	BMEII1083	206	186	D									O	190*	190*	190*	190*								Acts on Mot B
Master regulator	FtcR	BMEII0158		227																						

Ψ	All genes in the species are pseudogenized, or pseudogenized in this specific genome.
Ψ*	Some of the genes in this species are full length, some are pseudogenized.
D	All genes in species have the same internal deletion.
O	missing from genome
$\#^{M}$	An upstream mutation prevents full length gene.
289*	*B. suis* bv 5 has combined FlbT and FlbD into one long protein.
190*	All *B. canis* and almost all *B. suis* have a mutation that extends the length of MotE 4 amino acids to 190. Exceptions are bv 5 (missing) and strain S2-30 that does not have the mutation and the protein is the normal length.
